# Proteome-wide analysis of *Anopheles culicifacies* mosquito midgut: new insights into the mechanism of refractoriness

**DOI:** 10.1186/s12864-018-4729-3

**Published:** 2018-05-08

**Authors:** Sonam Vijay, Ritu Rawal, Kavita Kadian, Jagbir Singh, Tridibesh Adak, Arun Sharma

**Affiliations:** 1 Division of Protein Biochemistry and Structural Biology, National Institute of Malaria Research (ICMR), Sector 8, Dwarka, New Delhi, India; 2 Vector Biology Divisions, National Institute of Malaria Research (ICMR), Sector 8, Dwarka, New Delhi, India

**Keywords:** *Anopheles culicifacies*, Refractory, Midgut, Shot gun proteomics, iTRAQ, RT-PCR

## Abstract

**Background:**

Midgut invasion, a major bottleneck for malaria parasites transmission is considered as a potential target for vector-parasite interaction studies. New intervention strategies are required to explore the midgut proteins and their potential role in refractoriness for malaria control in *Anopheles* mosquitoes. To better understand the midgut functional proteins of *An. culicifacies* susceptible and refractory species, proteomic approaches coupled with bioinformatics analysis is an effective means in order to understand the mechanism of refractoriness. In the present study, an integrated in solution- in gel trypsin digestion approach, along with Isobaric tag for relative and absolute quantitation (iTRAQ)–Liquid chromatography/Mass spectrometry (LC/MS/MS) and data mining were performed to identify the proteomic profile and differentially expressed proteins in *Anopheles culicifacies* susceptible species A and refractory species B.

**Results:**

Shot gun proteomics approaches led to the identification of 80 proteins in *An. culicifacies* susceptible species A and 92 in refractory species B and catalogue was prepared. iTRAQ based proteomic analysis identified 48 differentially expressed proteins from total 130 proteins. Of these, 41 were downregulated and 7 were upregulated in refractory species B in comparison to susceptible species A. We report that the altered midgut proteins identified in naturally refractory mosquitoes are involved in oxidative phosphorylation, antioxidant and proteolysis process that may suggest their role in parasite growth inhibition. Furthermore, real time polymerase chain reaction (PCR) analysis of few proteins indicated higher expression of iTRAQ upregulated protein in refractory species than susceptible species.

**Conclusion:**

This study elucidates the first proteome of the midguts of *An. culicifacies* sibling species that attempts to analyze unique proteogenomic interactions to provide insights for better understanding of the mechanism of refractoriness. Functional implications of these upregulated proteins in refractory species may reflect the phenotypic characteristics of the mosquitoes and will improve our understandings of blood meal digestion process, parasite vector interactions and proteomes of other vectors of human diseases for development of novel vector control strategies.

**Electronic supplementary material:**

The online version of this article (10.1186/s12864-018-4729-3) contains supplementary material, which is available to authorized users.

## Background

According to World Health Organization (WHO), India accounts for 75% of total malaria cases among South East Asia region [1]. Female mosquitoes of the genus *Anopheles* are the major protagonists of malaria transmission. Among various *Anopheles* species, *Anopheles culicifacies* is considered as one of major malaria vector responsible for transmitting nearly 65% of total malaria cases in India [2]. This rural malaria vector species has been characterized as a sibling species complex with five sub species designated as A, B, C, D and E. All these sibling species are phylogenetically indistinguishable and are discriminated on the basis of vectorial capacity and malaria transmission rate [3]. A natural sub species of *An. culicifacies,* species B was detected and isolated from a specific area that is found to be refractory (poor vector) against human malaria parasites *Plasmodium vivax* (100% refractory) and *Plasmodium falciparum* (partially) [4]. Both potent and poor vector species however, display same distribution and adaptation to environment with similar zoophilic feeding preferences for human and cattle [5].

During malaria transmission, parasite in the mosquito midgut evades active mosquito immune responses to complete its life cycle but only few ookinetes thrive and traverse the midgut epithelium [6]. Hence, *Plasmodium* entry into the mosquito midgut epithelium is a major bottleneck point for its survival and development [7, 8]. This natural hindrance of parasite numbers in the midgut during invasion may be due to intervention of mosquito vector protective mechanisms or various encoded barriers [9, 10]. It is known that inhibition of parasite growth and development is either by ookinetes lysis or melanotic encapsulation in refractory mosquitoes [11–13]. Previous studies have also shown the various mechanisms operating in the midgut that may responsible for refractoriness in *An. culicifacies* [14–16]. Although, the phenomenon of natural refractoriness in mosquitoes is known, it is still not clear why some mosquitoes are susceptible and why some mosquitoes are able to resist infection [17–19].

It has been known that refractoriness is controlled by dominant genetic traits that may manifest by killing off the parasites in the midgut [20]. The co-evolution of different sibling species suggests a role of specific conserved proteins that may help to deal with biological changes occurring during parasite invasion in the midgut. In this perspective, very little is known about evolutionary divergence of *An. culicifacies* sibling mosquito species as the genome has not yet been sequenced. However, few proteomics studies on *An. culicifacies* species A salivary gland has been published [21]. Therefore, in order to unravel the molecular mechanism of refractoriness, it is vital to explore such mosquito factors in the midgut which may be responsible for imparting natural refractoriness in refractory *Anopheles* mosquito. The mosquito innate immunity may play a role by inactivation/activation of certain genes leading to expression and annotations of specific proteins known to contribute to parasites killing in refractory species.

In the present study, we combined shot gun- bottom up proteomic approach with iTRAQ labeling, data mining and validation by RT-PCR to identify midgut proteome and differentially expressed proteins in midguts of *An. culicifacies* susceptible species A and refractory species B. This is the first step in understanding of protein composition of mosquito midgut and first study that deepens our understanding about the changes in the midgut profile among both the potent and poor vector species. This in turn helps to explain molecular insights into differential vector competence, leading to the mechanism of refractoriness.

## Results

### Midgut proteome characterization

**Fig. 1:**
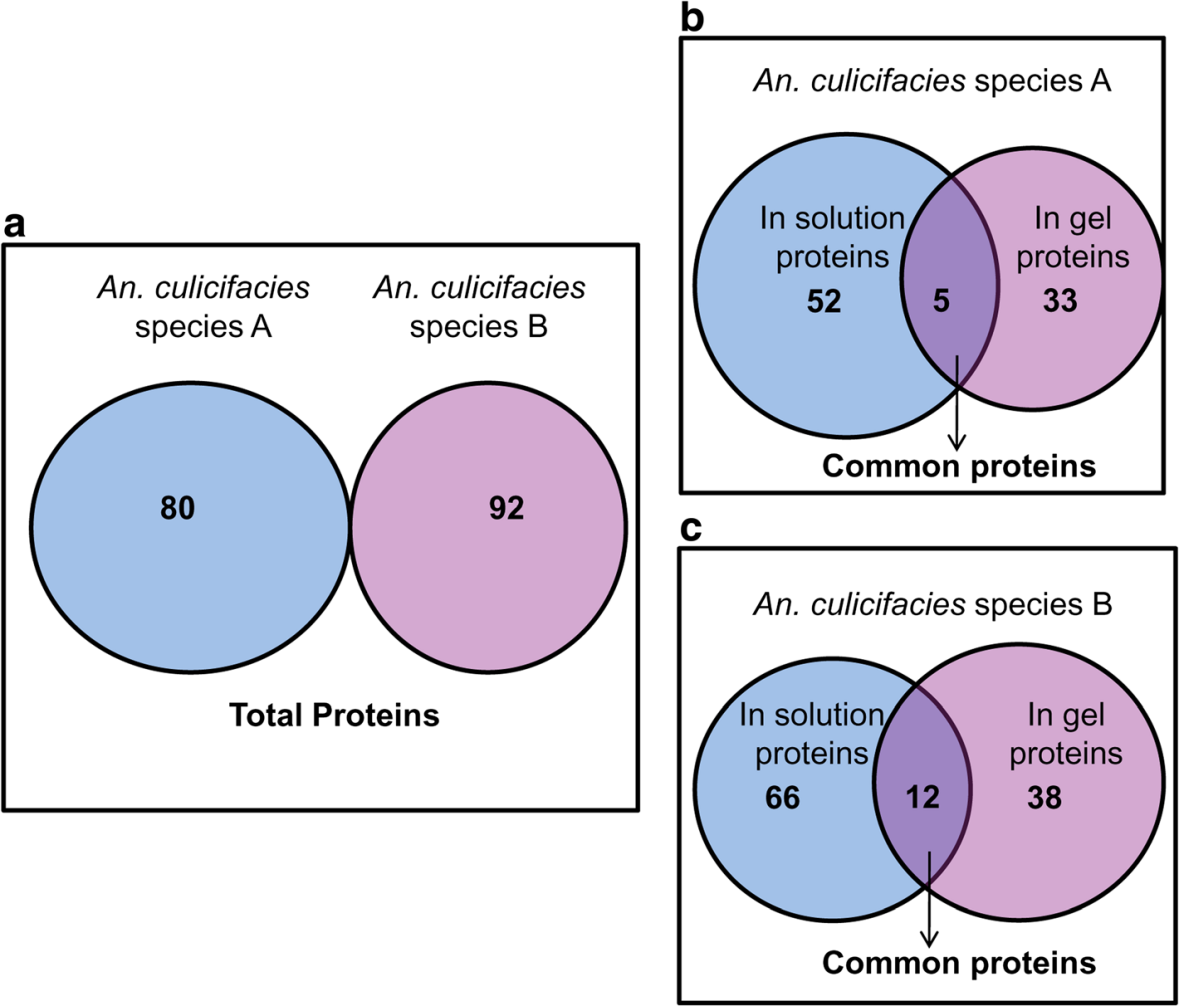
Representation of total functional proteins identified by in solution and in gel approach **a** Total identified functional proteins in *An. culicifacies* species A and species B. **b** Identified proteins in *An. culicifacies* species A. **c** Identified proteins in *An. culicifacies* species B

Since *An. culicifacies* sibling species genome have not been sequenced yet hence proteomics investigation was performed for the comprehension of midgut proteins and their molecular function in both susceptible and naturally refractory species. Using the LC/MS/MS analysis and SEQUEST HT algorithm, a total of 80 functional putative proteins were identified in midgut of *An. culicifacies* susceptible species A and 92 proteins in midgut of *An. culicifacies* refractory species B with a false discovery rate of 0.01 (Fig. 1a). These cataloging of proteins were prepared from the common proteins identified among the replicates. Total midgut proteins identified using different approaches i.e. in solution and in gel trypsin digestion in *An. culicifacies* species A and species B respectively are shown (Fig. 1b and c).

**Fig. 2:**
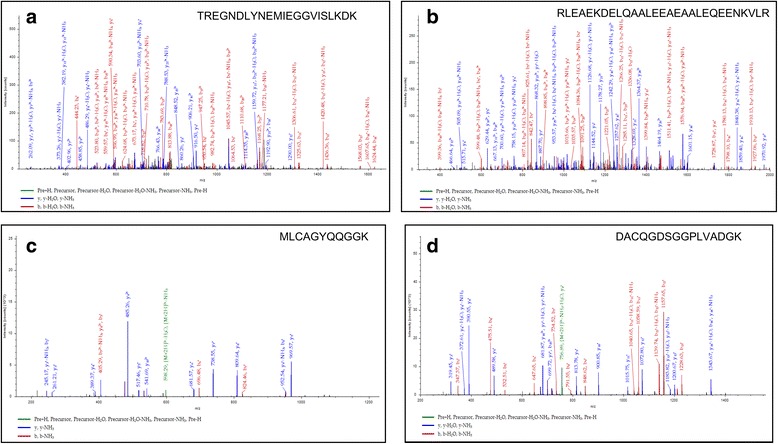
Representation of peak spectrum of *An. culicifacies* species A and species B analyzed by in solution digestion approach followed by LC/MS/MS. **a-b** (Species A) **a** MS/MS spectrum of product ion at m/z 828 corresponds to the peptide sequence TREGNDLYNEMIEGGVISLKDK, matched to the Myosin (AGAP010147) (132 min) **b** MS/MS spectrum of peptide sequence RLEAEKDELQAALEEAEAALEQEENK at m/z 986, matched to the ATP synthase subunit beta protein peak (196 min).**c-d** (Species B) **c** MS/MS spectrum of product ion at m/z 607 corresponds to the peptide sequence MLCAGYQQGGK (46 min) and **d** Spectrum of product ion at m/z 774 corresponds to the peptide sequence DACQGDSGGPLVADGK (36 min) matched to the Trypsinogen precursor of ANTRYP7 protein. Red color indicates *b* ions, blue color indicates *y* ions

Among identified total 52 significant putative proteins using in solution trypsin digestion in *An. culicifacies* species A, Myosin (AGAP010147) was identified with highest score (2730) and highest peptide matches (48 peptides) with 24% sequence similarity. The protein with maximum sequence coverage was found to be ATP synthase subunit beta (57%) with total 21 peptides matches. Their representative MS/MS spectrum of single peptide at respective m/z were shown (Fig. 2a and b). A detailed summary of all the identified putative proteins according to the molecular function i.e. cytoskeletal proteins, proteins involved in energy production, binding, glycolysis, redox mechanism, immune related proteins, transport***,*** signal transduction were shown (Additional file 1: Table S1). In *An. culicifacies* species B, a total of 66 significant putative proteins were identified using in solution trypsin digestion approach. A total ion chromatogram of species B and representative MS/MS spectrum of Trypsinogen precursor of ANTRYP7 protein peptide are shown (Fig. 2c and d). A detailed list of functional putative protein with categories like Immune related proteins, proteins involved in glycolytic metabolism, structural component, cell differentiation, protein as a receptor depicted with their, sequence coverage; peptide matches, molecular weight, pI are shown (Additional file 2: Table S2).

**Fig. 3:**
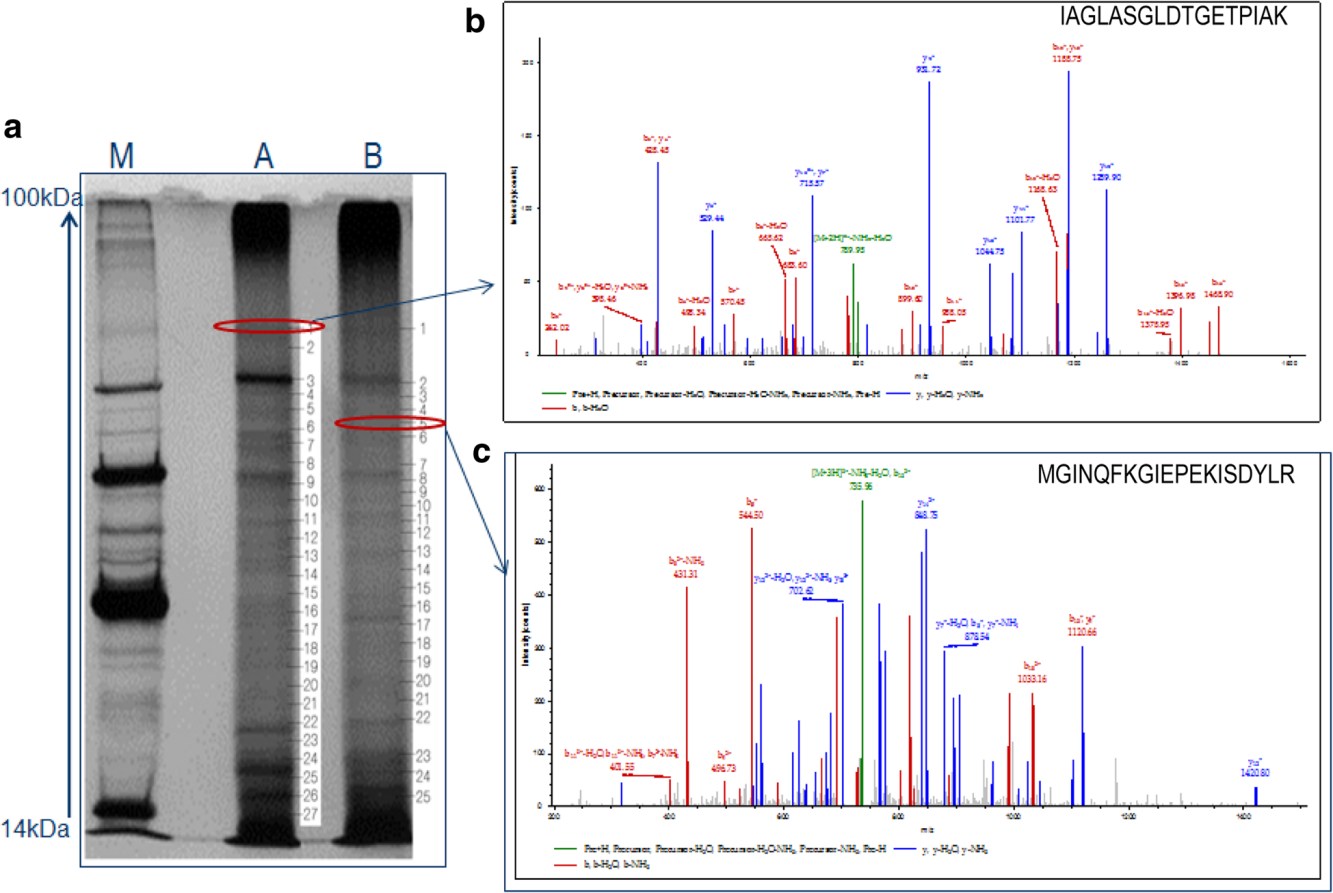
SDS Page and MS/MS spectrum of proteins peptide sequences. **a** 1-D gel of the midgut extract of *An. culicifacies* species A (27 protein Bands, Lane A), *An. culicifacies* species B (25 protein Bands, Lane B) and molecular weight markers (Lane M). **b** MS/MS spectrum of peptide sequence IAGLASGLDTGETPIAK of Sodium/potassium transporting ATPase alpha chain protein at m/z 808 identified from Band No. 1 of *An. culicifacies* species A. **c** MS/MS spectrum of peptide sequence MGINQFKGIEPEKISDYLR (m/z 757) of Guanine nucleotide binding protein identified from Band No. 5 of *An. culicifacies* species B

Further from in gel digestion approach, protein banding patterns revealed both similarities and differences between species A (33 proteins corresponds to 27 bands) and species B (38 proteins corresponds to 25 bands) (Fig. 3). All these putative proteins of both species A and species B as per their gel band numbers, sequence coverage and molecular functions were shown respectively (Additional file 3: Table S3 and Additional file 4: Table S4). Representative example of chromatogram peak of peptide sequence of ATPase alpha chain protein identified from band 1 (IAGLASGLDTGETPIAK) in species A (Fig. 3a) and Guanine nucleotide binding protein identified from band 5 in species B were shown (Fig. 3b).

### Biological role of putative functional proteins

**Fig. 4:**
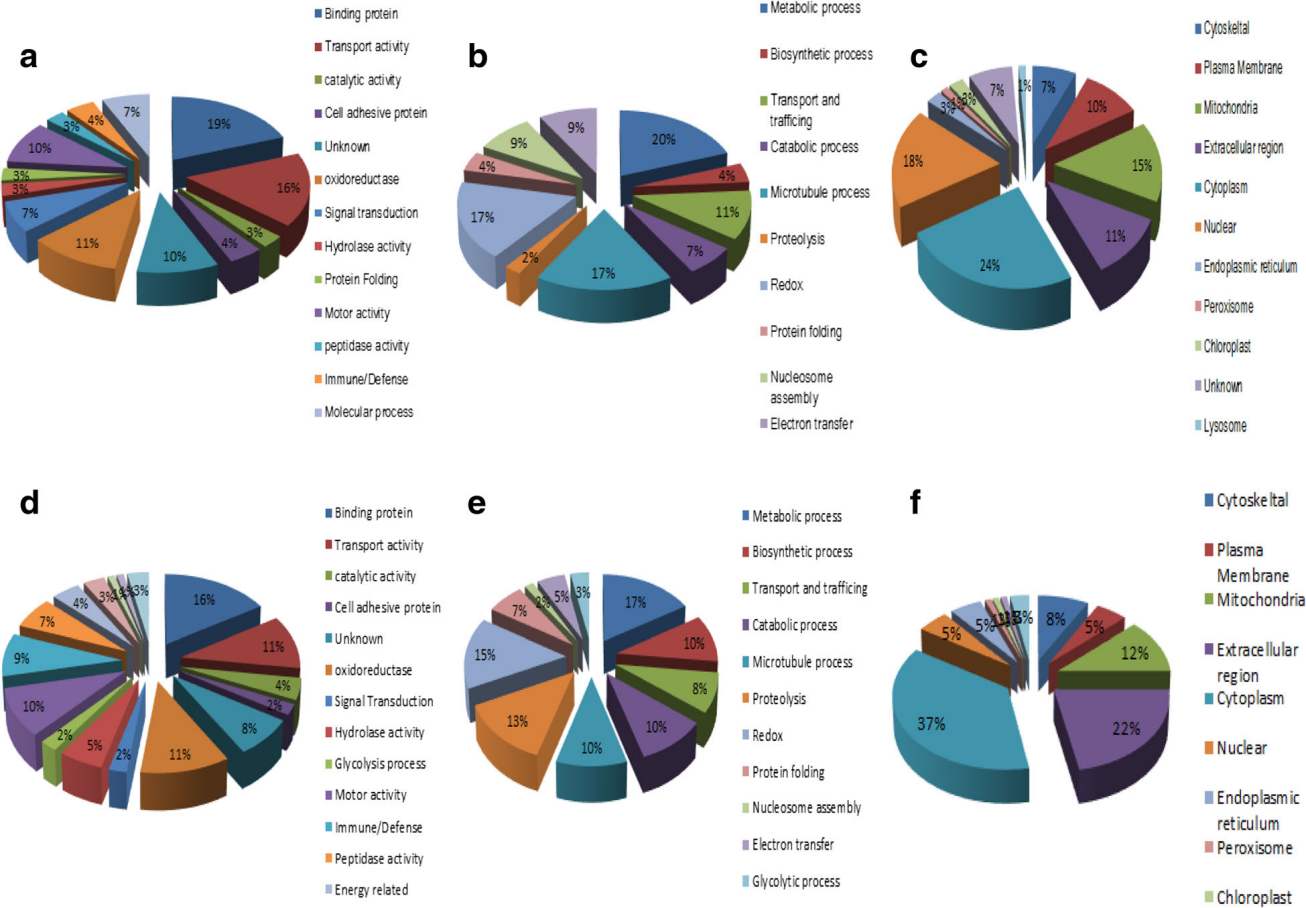
Illustration of total identified midgut proteins of *An. culicifacies* species A and *An. culicifacies* species B. **a-c (**Species A) **a** Molecular function **b** Biological process **c** Intracellular localization. **d-f** (Species B) **d** Molecular function **e** Biological process **f** Intracellular localization. The percentage of total identified proteins is depicted in pie chart

Intracellular localization, biological process and functional classification of all putative proteins of *An. culicifacies* susceptible species A and *An. culicifacies* refractory species B were performed. In *An. culicifacies* species A, our study reveals that most of the proteins on the basis of molecular function were sorted under category of Binding proteins (19%), Transport protein (16%), Oxi-redox activity (11%), Signal transduction (10%) (Fig. 4a). Most of the putative proteins associated with biological processes were clustered in metabolic process (20%), microtubular process (17%), redox process (17%) and transport (11%) (Fig. 4b). Further on the basis of cellular localization most of proteins in *An. culicifacies* susceptible species A were found to be located in the cytoplasm (24%), nucleus (18%), mitochondria (15%), extracellular region (11%), plasma membrane (10%) (Fig. 4c). In refractory species B, proteins according to molecular function were mainly categorized in same group as in species A (Fig. 4d). Most of proteins on the basis of biological process were sorted under metabolic process (17%), redox process (15%), proteolysis (13%), microtubule (10%) (Fig. 4e). Further in *An. culicifacies* refractory species, contrary to susceptible species most of the proteins were found to be localized at extracellular region (22%) after cytoplasm (37%) instead of nuclear region (5%) (4f).

### Quantitative differential proteomics- iTRAQ labeling approach

Quantitative proteomics was performed among susceptible and refractory *An. culicifacies* midgut for differential expression analysis. iTRAQ labeling followed by LC/MS/MS has generated raw data from three fractions (250 mM, 350 mM and 450 mM SCX fraction) that were searched using Proteome Discoverer 1.4 and analyzed by both SEQUEST and MASCOT algorithm against UniProt/NCBInr mosquito database.

**Fig. 5:**
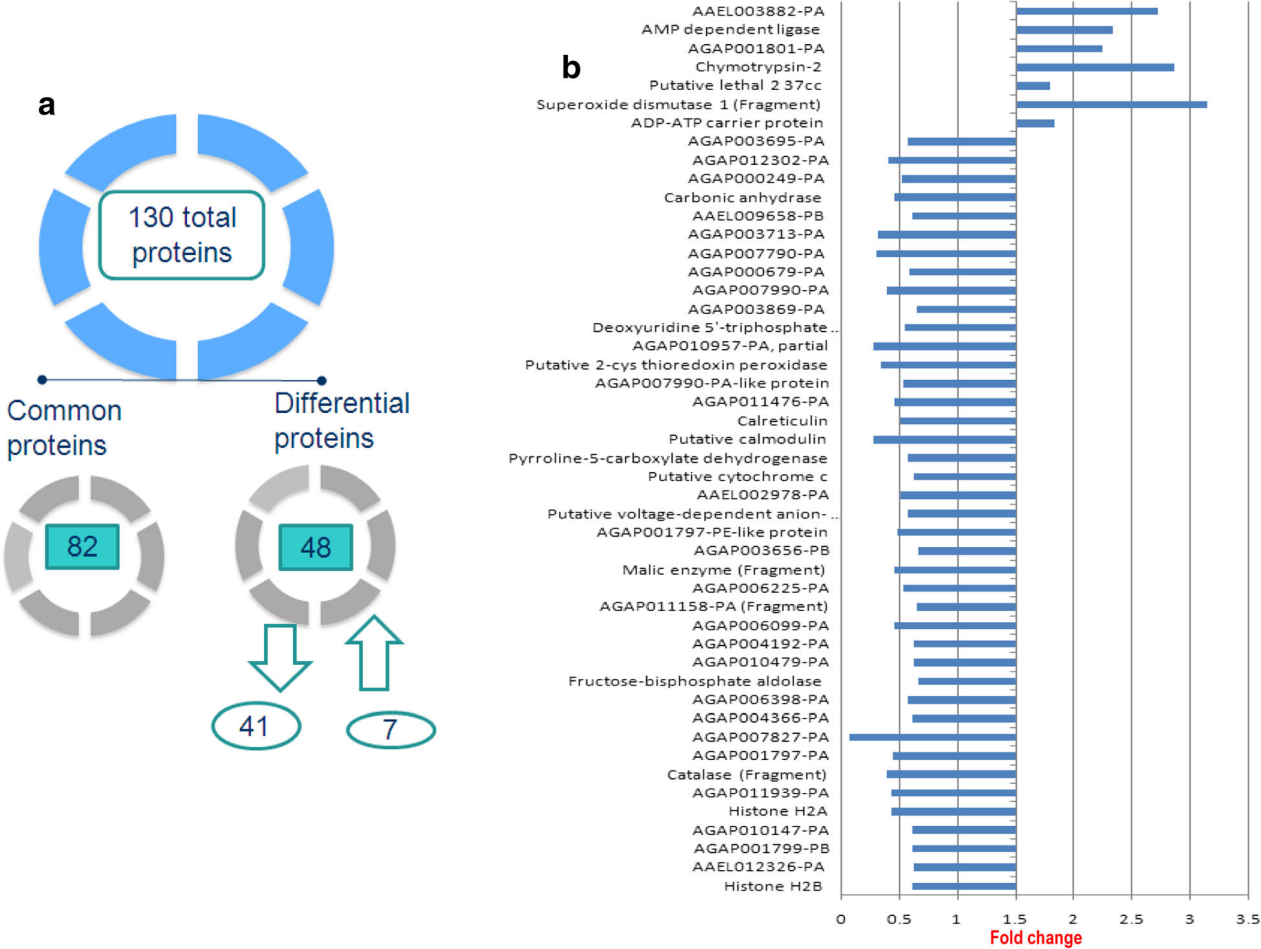
Depiction of iTRAQ analyzed proteins. **a** Representation of total identified iTRAQ analyzed proteins **b** Total 48 differentially expressed proteins identified in *An. culicifacies* refractory species B with respective fold changes

Using iTRAQ ratio criteria, 48 proteins were found to be differentially expressed from the total 130 proteins identified across the *An. culicifacies* midgut. Of these, 41 proteins were downregulated, 7 proteins were upregulated in refractory species B in comparison to susceptible species A and 82 proteins were equally expressed between both species A and species B (Fig. 5a). These identified differentially expressed proteins in *An. culicifacies* species B along with their fold change are shown (Fig. 5b). Depiction of upregulated and downregulated proteins in refractory species as compared to susceptible species with their peptide sequence/number, coverage, iTRAQ ratios are shown respectively (Tables 1 and 2). Equally expressed proteins present in both *An. culicifacies* refractory and susceptible with detailed information’s were also provided (Additional file 5: Table S5).

**Table 1:** A catalogue of identified upregulated proteins using iTRAQ labeling method in refractory *An. culicifacies* species B in comparison to susceptible *An. culicifacies* species A

S.no	Uniprot no.	Protein	∑ coverage	Peptide Sequence	M.wtkDa	iTRAQ ratio	Function
1	Q27238	ADP-ATP carrier protein (similar to *An. gambiae*)	9	TAVAPIER GNLANVIR LGADVGR GMLPDPK	40.3	1.83	Transporter activity/energy metabolism
2	Q6RBZ4	Superoxide dismutase 1 (Fragment) (similar to *An. gambiae*)	8	SDPSAELQK NLSPDR	22.9	3.14	Ion binding, Detoxification
3	A0A023EPD0	Putative lethal 2 37cm^3^ (similar to *Aedes albopictus*)	6	VSDDLTER NVPVVTGSK	33.5	1.79	Prohibitin protein/ Immune response
4	Q17025	Chymotrypsin-2 (similar to *An. gambiae*)	5.04	EGGELLK LTGWGR	27.9	2.859	serine-type endopeptidase activity
5	Q7PY94	AGAP001801-PA (similar to *An. gambiae*)	5	HNQYPK MRPLCSK	29.5	2.240	Unknown
6	B0X9E3	AMP dependent ligase (similar to *Culex quinquefasciatus*)	5	AAQNLTK IFVMENAPNEECAVDQLFR	59.8	2.331	catalytic activity
7	Q17E94	AAEL003882-PA (similar to *Aedes aegypti*)	3	ENELLK ELRLELEDCKQEMAQAR LELEDCKQEMAQAR QIATLEER DLRELR EIEQLEER RERNEAVNER	258	2.717	Unknown

**Table 2:** A catalogue of downregulated proteins identified using iTRAQ labeling method *An. culicifacies* in species B

S.no	UniProt no.	Protein	∑ coverage	Peptides	M.wt (kDa)	Function	Ratio
1.	B0WZ30	Histone H2B (similar to *Culex quinquefasciatus*)	32	5	13.8	DNA binding	0.61
2.	Q1HQX3	AAEL012326-PA (similar to *Aedes aegypti*)	31	6	16.8	calcium ion binding	0.62
3.	F5HME4	AGAP001799-PB (similar to *Anopheles gambiae)*	24	8	32.4	Unknown	0.61
4.	Q7PJV2	AGAP010147-PA (similar to *Anopheles gambiae)*	23	56	224.2	ATP binding, motor activity	0.60
5.	B0X773	Histone H2A (similar to *Culex quinquefasciatus)*	20	4	13.3	DNA binding	0.43
6.	Q5TMR7	AGAP011939-PA (similar to *Anopheles gambiae)*	20	7	35.9	catalytic activity	0.43
7.	Q1HRH7	Catalase (Fragment) (similar *to Aedes aegypti*)	20	8	48.5	catalytic activity	0.39
8.	Q380N3	AGAP001797-PA (similar to *Anopheles gambiae*)	17	17	84.3	Unknown	0.44
9.	Q7Q3D8	AGAP007827-PA (similar to *Anopheles gambiae*)	15	10	46.6	phosphopyruvate hydratase activity	0.068
10.	Q7QA89	AGAP004366-PA (similar to *Anopheles gambiae*)	11	8	63.5	oxidoreductase activity	0.60
11.	Q7Q5J5	AGAP006398-PA (similar to *Anopheles gambiae*)	10.34	3	31.2	unknown function	0.56
12.	F5HKV6	Fructose-bisphosphate aldolase (similar to *Anopheles gambiae*)	9.64	4	39.2	aldolase activity	0.66
13.	Q7QDY3	AGAP010479-PA (similar to *Anopheles gambiae*)	7.86	4	45.0	unknown	0.62
14.	Q7PQK5	AGAP004192-PA (similar to *Anopheles gambiae*)	7.13	6	72.7	Protein folding	0.62
15.	Q7Q609	AGAP006099-PA (similar to *Anopheles gambiae*)	6.61	4	45.1	metal ion binding, catalytic activity	0.45
16.	Q7QHE7	AGAP011158-PA (Fragment) (similar to *Anopheles gambiae*)	6.15	3	51.5	pyridoxal phosphate binding	0.64
17.	Q7Q5T1	AGAP006225-PA (similar to *Anopheles gambiae*)	5.14	8	138.7	oxidoreductase activity	0.53
18.	T1DPT6	Malic enzyme (Fragment) (similar to *Anopheles aquasalis)*	4.43	3	79.2	metal ion binding	0.45
19.	F5HLD4	AGAP003656-PB (similar to *Anopheles gambiae*)	4.12	18	450.9	protein binding, Immunoglobulin-like domains	0.65
20.	A0A084VWP1	AGAP001797-PE-like protein (similar to *Anopheles sinensis*)	17	6	41.657	tropomyosin	0.48
21.	T1DN92	Putative voltage-dependent anion-selective channel (similar to *An. aquasalis*)	8	2	38.131	voltage-gated anion channel activity	0.57
22.	Q17GL0	AAEL002978-PA (similar to *Aedes aegypti*)	10	5	65.476	aminopeptidase activity	0.5
23.	A0A023EEY5	Putative cytochrome c (similar to *Aedes albopictus*)	21	2	16.3	electron carrier activity	0.62
24.	B0WKF4	Pyrroline-5-carboxylate dehydrogenase (similar to *Culex quinquefasciatus)*	10	6	77.7	oxidoreductase activity	0.56
25.	A0A023EGV4	Putative calmodulin (similar to *Aedes albopictus*)	25	5	19.538	calcium ion binding	0.27
26.	A0A084VKA2	Calreticulin (similar to *Anopheles sinensis*)	7	4	59.369	calcium ion binding	0.5
27.	Q7Q343	AGAP011476-PA (similar to *Anopheles gambiae*)	7	5	105.89	amidase activity	0.45
28.	A0A084WD61	AGAP007990-PA-like protein (similar to *Anopheles sinensis*)	4	3	655.68	glucuronosyl transferase activity	0.53
29.	A0A023EJ61	Putative 2-cys thioredoxin peroxidase (similar to *Aedes albopictus*)	9	2	267.66	peroxidase activity	0.33
30.	Q7QGY7	AGAP010957-PA, partial (similar to *Anopheles gambiae*)	24	5	199.5	calcium ion binding	0.27
31.	B0WED4	Deoxyuridine 5′-triphosphate nucleotidohydrolase(similar to *Culex quinquefasciatus)*	21	3	15.9	Diphosphatase activity	0.543
32.	Q7QFS4	AGAP003869-PA (similar to *Anopheles gambiae*)	11.29	6	54	Aminopeptidase activity	0.646
33.	Q7Q3R0	AGAP007990-PA (similar to *Anopheles gambiae*)	10.22	6	61.4	transferase activity	0.380
34.	Q7QEF5	AGAP000679-PA (similar to *Anopheles gambiae*)	9	4	46.4	Aminoacylase activity	0.585
35.	A0NFA5	AGAP007790-PA (similar to *Anopheles gambiae*)	7.32	4	35.5	Ion transport	0.291
36.	A0NDL8	AGAP003713-PA (similar to *Anopheles gambiae*)	6.84	1	12.8	Unknown	0.316
37.	Q16V79	AAEL009658-PB (similar to *Aedes aegypti*)	6.84	4	66.9	Alpha trehalase activity/ catalytic activity	0.606
38.	B0W447	Carbonic anhydrase (similar to *Culex quinquefasciatus*)	5.76	2	31.5	carbonate dehydratase activity	0.450
39.	Q7QF10	AGAP000249-PA (similar to *Anopheles gambiae*)	4.30	4	69.7	mannosyl-glycoprotein endo-beta-N-acetyl glucosaminidase activity	0.520
40.	Q7PV84	AGAP012302-PA (similar to *Anopheles gambiae*)	3.66	2	71.1	sulfate transmembrane transporter activity	0.405
41.	Q7QAH7	AGAP003695-PA (similar to *Anopheles gambiae*)	3.48	5	105.5	metallopeptidase activity	0.577

### Validation of iTRAQ data using real time PCR

**Fig. 6:**
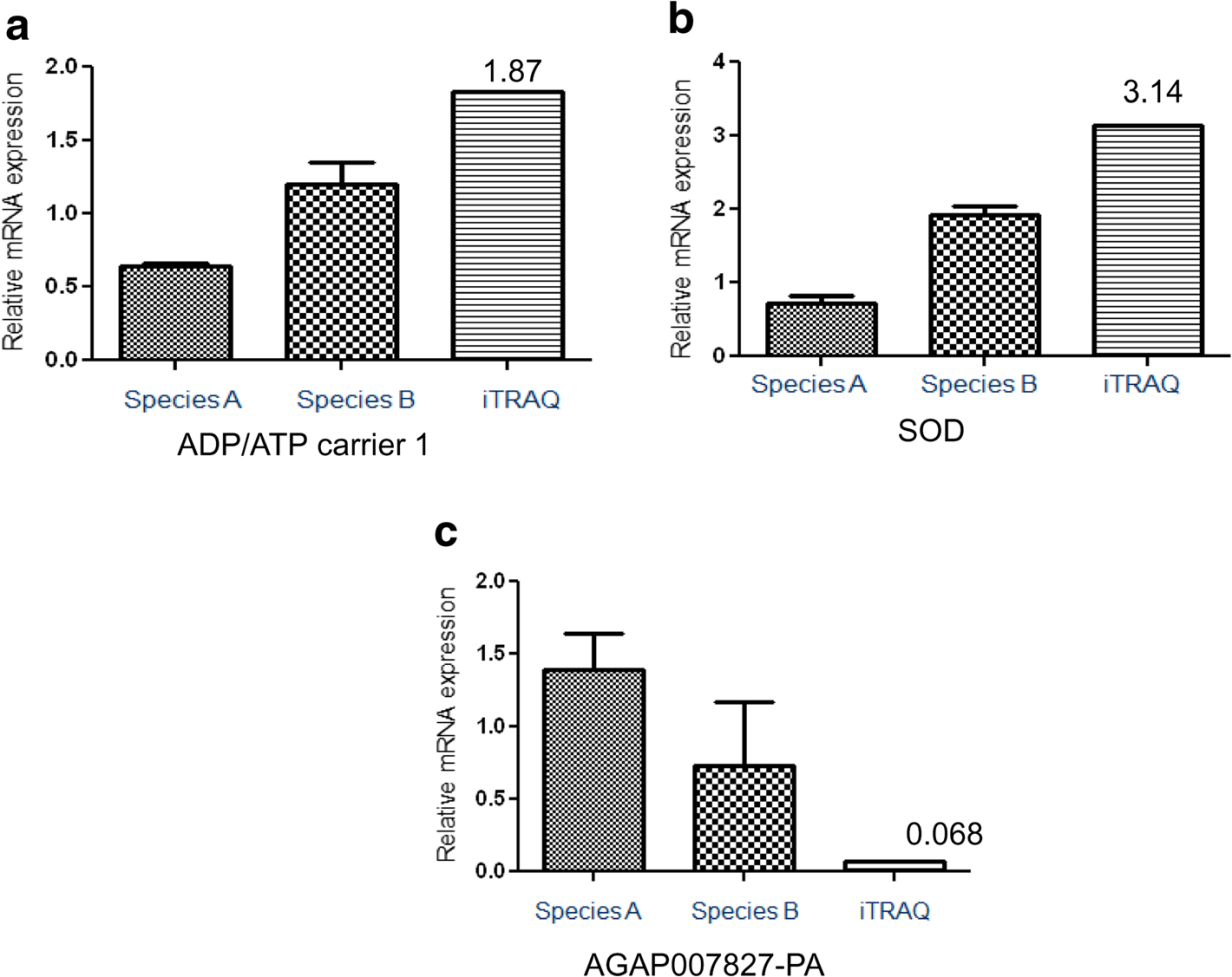
Validation of iTRAQ results through RT-qPCR. **a** Up regulated relative mRNA expression level of ADP/ATP carrier 1 protein [*p* = 0.031, *p* < 0.05, Student t- test] **b** SOD protein [*p* = 0.017] **c** Down regulated mRNA expression of AGAP007827-PA (Enolase) [*p* = 0.04] in refractory *An. culicifacies* species B than susceptible *An. culicifacies*

For evaluation of correlation between mRNA expression level and protein abundance, we chose higher scoring upregulated protein ADP/ATP carrier 1, Superoxide dismutase (SOD) and one downregulated protein AGAP007827-PA (0.068, low iTRAQ ratio). The relative mRNA expression level of ADP/ATP carrier 1 protein and SOD were found to be higher in refractory species B with approx. 2 fold higher in comparison to susceptible species A (*p* = 0.03; *p* = 0.017 respectively) (Fig. 6a and b). Similarly approx. 2 fold higher expression of AGAP007827-PA (phosphopyruvate hydratase activity) protein was found in susceptible species as compared to refractory species (*p* = 0.04) (Fig. 6c).

### Protein-protein interaction and pathways analysis

**Fig. 7:**
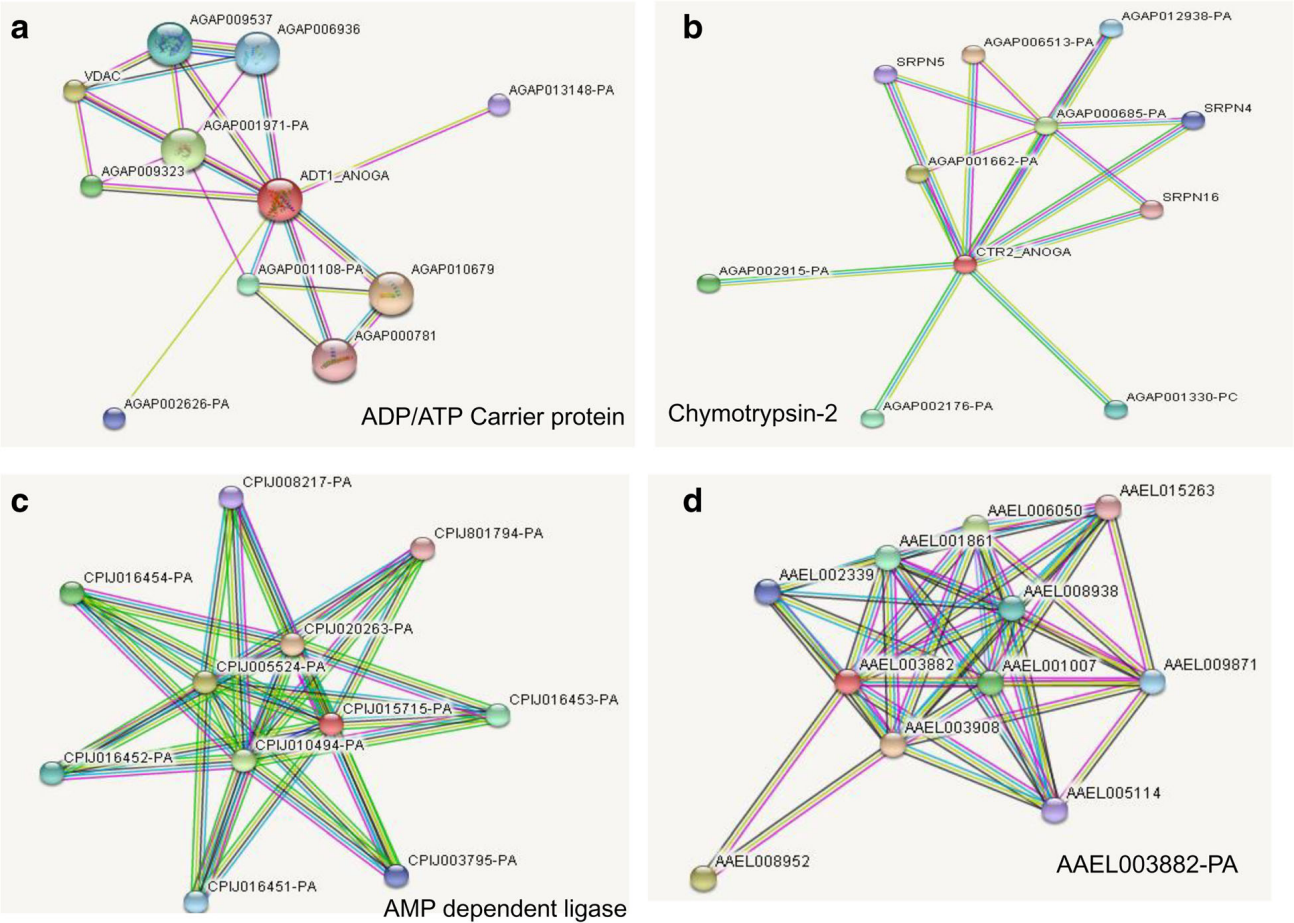
Protein –protein interactions analysis of upregulated proteins using String network. **a** STRING network analysis between ADP/ATP carrier 1 protein and other associated proteins with 22 edges and various evidences**. b** STRING network analysis of Chymotrypsin protein. **c** STRING network analysis of AMP dependent ligase enzyme. d STRING network analysis between identified unknown AAEL003882-PA protein

iTRAQ analyzed upregulated and few downregulated proteins were inputted into Search Tool for the Retrieval of Interacting Genes/Proteins (STRING) web tool for exploring protein–protein interactions. These analyses were performed in order to determine the relationship of these important proteins with other proteins located in a network hub. Among the available network, first network of ADP/ATP carrier 1 protein involves the functional association of 11 nodes that have relationship with voltage dependent anion channel protein, Ubiquitin, Prohibitin, cytochrome C, Nicotinamide adenine dinucleotide (NAD) dependent protein deacetylase and serine type endopeptide activity. All these proteins connecting with some lines and thickness of these lines specified the strength of interactions. They implied that these interacting proteins are mainly involved in electron carrier activity, protein transport, binding and regulation and played important functions (Fig. 7a). For second network of chymotrypsin 2, functional association network of 11 nodes were found with serine-type endopeptidase inhibitor activity, calcium ion binding, and serine-type endopeptidase activity (Fig. 7b). Another protein, AMP dependent ligase was found to be associated with network of Acetyl-coa carboxylase, Acyl-coa dehydrogenase, crotonobetainyl- coa dehydrogenase, oxidoreductase, fatty acid oxidation complex subunit. These interactions indicated to be involved in catalytic activity, ATP binding, ligase activity, oxidation-reduction process (Fig. 7c). Functional association network of one hypothetical enzyme (AAEL003882-PA) interpreted to be associated with nuclear pore complex protein, Sentrin/sumo-specific protease, Nuclear RNA export factor 2, RNA and export factor binding protein and conserved hypothetical protein (Fig. 7d). For downregulated proteins we tried to link all proteins in a network hub however, we found mainly low confidence protein - protein interactions of available network. Merely few proteins that showed the association with high confidence were mainly involved in arginine-proline metabolism and metabolic pathways.

Kyoto Encyclopedia of Genes and Genomes (KEGG) pathway database was used for pathway enrichment analysis. All upregulated and downregulated proteins were analyzed and a total of 20 proteins were identified with pathways that were classified into 10 categories. These categories were Phototransduction-fly, Starch and Sucrose metabolism, Glycolysis / Gluconeogenesis, Biosynthesis of Amino Acids, Protein processing in Endoplasmic Reticulum, Oxidative Phosphorylation, Metabolic pathways, Pyrimidine and Purine metabolism, Fatty acid metabolism and RNA transport (Table 3).

**Table 3:** KEGG pathways of differentially expressed proteins identified in *An. culicifacies* refractory species B

S.no	KEGG ID	Pathway	Expression
1	04745	Phototransduction-fly	
Proteins (3)		AAEL012326-PA (similar to *Aedes aegypti*)	Down
		AGAP010147-PA (similar to *Anopheles gambiae*)	Down
		AGAP010957-PA, partial (similar to *Anopheles gambiae*)	Down
2	00500	Starch and sucrose metabolism	
Proteins (2)		AGAP011939-PA (similar to *Anopheles gambiae*)	Down
		AAEL009658-PB (similar to *Aedes aegypti*)	Down
3	00010	Glycolysis / Gluconeogenesis	
Proteins (2)		AGAP007827-PA (similar to *Anopheles gambiae*)	Down
		Fructose-bisphosphate aldolase (similar to *Anopheles gambiae*)	Down
4	00330	Arginine and proline metabolism Biosynthesis of amino acids	
Proteins (5)		AGAP004366-PA (similar to *Anopheles gambiae*)	Down
		Pyrroline-5-carboxylate dehydrogenase (similar to *Culex quinquefasciatus*)	Down
		AGAP000679-PA (similar to *Anopheles gambiae*)	Down
		AGAP003695-PA (similar to *Anopheles gambiae*)	Down
		AAEL002978-PA (similar to *Aedes aegypti*)	Down
5	04141	Protein processing in endoplasmic reticulum	
Protein (1)		AGAP004192-PA (similar to *Anopheles gambiae*)	Down
6	00190	Oxidative phosphorylation	
Protein (2)		AGAP006099-PA (similar to *Anopheles gambiae*)	Down
		ADP/ATP carrier protein (similar to *An. gambiae*)	Up
7	00450	Metabolic pathways	
Protein (1)		AGAP011158-PA (Fragment) (similar to *Anopheles gambiae*)	Down
8	00230	Pyrimidine metabolism and purine metabolism	
Proteins (2)		AGAP006225-PA (similar to *Anopheles gambiae*)	Down
		Deoxyuridine 5′-triphosphate nucleotidohydrolase (similar to *Culex quinquefasciatus*)	Down
9	01212	Fatty acid metabolism	
Protein (1)		AMP dependent ligase (similar to *Culex quinquefasciatus*)	Up
10	03013	RNA transport	
Protein (1)		AAEL003882-PA (similar to *Aedes aegypti*)	Up

## Discussion

*Anopheles culicifacies* sibling species, a rural Indian malaria vector has a co-evolutionary history which is important for malaria epidemiology. Therefore, despite the co-evolution of sibling species, vector-parasite interactions in species B have rendered these mosquitoes poor vector at population level. A critical biochemical, molecular and immunological interactions occur in the mosquito midguts during parasite invasion which act as an important barrier for parasite development and hence it is a promising target for blocking *Plasmodium* transmission. Thus, the identification of various expressed and annotated protein factors that may be responsible for the inhibition of the parasite growth and development is of utmost importance. In our effort to advance knowledge about refractoriness, we have combined shot gun proteomics along with iTRAQ with data mining to analyze midgut proteome and differential expressed proteins in *An. culicifacies* species A and species B.

In the present study, the shot gun approach helps in merging in-solution and in-gel proteomic dataset into a comprehensive catalogue which revealed various proteins that may reflect the phenotypic response in the mosquitoes. Though not as expected, less number of putative proteins among sibling species were identified which may be due to the fact that the genome (and the subsequent prediction of its protein complement) of *An. culicifacies* is not available. As this approach is meant for qualitative analysis rather than comparative analysis however, in this initial study gene ontology (GO) analysis indicates the presence of more number of proteolytic and peptidases proteins in refractory species than susceptible species. Examples of proteolytic proteins are serine proteases, Trypsin, Trypsin like serine proteases, chymotrypsinogen-like protease, serpins, many proteins with serine-type endopeptidase activity and metallopeptidases etc. These are the main enzymes known to be involved in food digestion and also key mediators of host vector- parasite interactions like aminopeptidases [22]. Expression of these multi gene family of tryspin like serine protease are biphasic that means some are constitutively expressed and some after blood meal [23, 24]. Higher number of serine proteases in refractory species B may indicate their involvement in the immune responses that amplify the signal modulation leading to the activation of melanization reactions [25]. Recently, one study has identified the expression of putative serine peptidases at protein level in the midgut of sugar fed *An. aquasalis* females mosquitoes [26]. Proteolytic enzyme serpins are important immunomodulators that may be able to activate the cytoprotective mechanisms in the mosquito [27]. These serpins regulated prophenol oxidase activation and so regulates melanization process. Hence, findings of more number of proteolysis cascade enzymes in refractory species of *An. culicifacies* may be directly or indirectly affecting parasite growth and maturation.

To investigate the differentially expressed proteins among *An. culicifacies* sibling species, iTRAQ analysis was performed that revealed the up-regulation of 7 proteins. Among these ADP-ATP carrier protein is a mitochondria membrane protein which is associated with oxidative phosphorylation pathway (PPI enrichment, *p* = 0.001). Previous studies have shown the importance of mitochondria as a cellular source of reactive oxygen species (ROS) [28, 29]. To our knowledge very little description of ADP-ATP carrier protein was previously reported however strongest structural match of this protein with mitochondrial carrier 1 protein was known [30]. Functionally mitochondrial carrier 1 protein was known to modulate ROS production in *Anopheles gambiae*. It has been shown that silencing of mitochondrial carrier 1 gene promotes susceptibility to *Plasmodium* infection due to reduced ROS production and mitochondria membrane potential [28]. Hence we can predict that this ADP/ATP carrier 1 protein perhaps has same function as mitochondrial carrier 1 but knocking out this protein might give better insight into its possible response to *Plasmodium* infection. Validation by real time PCR also confirmed the higher expression of ADP-ATP carrier 1 protein in refractory species B as compared to susceptible species A.

SOD, another upregulated protein was identified to be involved in enhancing immunity and in limiting parasite infection. It mainly helps in detoxification of ROS that are potentially toxic to the host and hence protect the cells. It is interesting to note that by iTRAQ method upregulated SOD was found in the midgut of refractory species and on the other hand downregulated catalase enzyme was identified. Catalase enzyme, an antioxidant is known for detoxification of generated H_2_O_2_ produced by SOD to water and oxygen. Hence we hypothesize that this higher H_2_O_2_ levels due to lower expression of catalase may be responsible for inhibiting the parasite growth in midgut. Previous studies also showed the correlation of suppression of catalase expression in midgut of *P. berghei* infected mosquitoes with higher level of H_2_O_2._ It has been shown that higher H_2_O_2_ level is responsible for increased ookinetes lysis and reduce oocyst formation, therefore contributes to limit *Plasmodium* infection by lytic mechanism during their transit through midgut epithelium [31, 32]. Previous literatures supported the hypothesis that increased SOD levels lead to high H_2_O_2_ level as reduced catalase expression. This may employ an effective immune response mechanism in naturally available refractory species of *An. culicifacies*. Prohibitin, another upregulated protein is known for multifaceted role in cell physiology and participating in immune response mechanism but its function is still unknown in insect’s immunity [33]. In previous studies its role is known in mosquitoes as a receptor for dengue virus infection and in viral susceptibility [34]. Two unknown proteins were also found to be upregulated; among them protein AAEL003882-PA was found to be associated with RNA transport (PPI enrichment, *p* = 9.33e-11). AMP dependent ligase involved in fatty acid metabolic pathway was also found to be upregulated in refractory species (PPI enrichment, *p* = 2.2e-09). This modulation of fatty acid metabolism could be a cellular mechanism to produce energy and also fight to inhibit the parasite development in midgut of refractory mosquito.

Among downregulated proteins in refractory species B, a Kreb cycle enzyme (a Malic enzyme), an antioxidant protein which catalyzes the conversion of L-malate to pyruvate and CO_2_ was identified. It uses NAD as a coenzyme and reversible oxidative decarboxylation produces reduced NADPH [35]. This generated NADPH helps in the detoxification of ROS [36]. We hypothesize that the downregulated expressions of both malic and catalase enzymes may cause accumulation of ROS in midgut of refractory species that might be toxic to growth of parasite. Interestingly enzymes of glycolysis (Fructose bisphosphate aldolase and enolase/phosphopyruvate hydratase) were also down regulated in species B and same expression pattern of Enolase enzyme was found at RNA level in refractory species. Few studies have shown the downregulated pattern of the energy metabolic enzymes in *Drosophila* [37]. It was also reported that aldolase enzymes indirectly or directly help in parasite invasion and hence important for infection progression [38]. These downregulated observations of glycolytic enzymes might explain the prevention of parasite infection modulation in refractory mosquito species or also to save energy. Role of another downregulated midgut protein i.e. calreticulin in the interaction with *Plasmodium* ookinetes surface proteins in *An. albimanus* [39] and *An. stephensi* [40] were demonstrated and suggesting a promising and novel transmission blocking vaccine target. Similarly, two important downregulated enzymes of glutathione metabolic pathways i.e. Alanine aminopeptidase and leucine aminopeptidase were also significant as malaria transmission-blocking vaccines target. These enzymes in the mosquito midgut were shown to be as a receptor for *Plasmodium* [41].

## Conclusions

Our study highlights the molecular framework underpinning the mechanism of refractoriness by identifying proteins that are uniquely expressed in *An. culicifacies* refractory species B, a poor vector of malaria transmission to humans. These identified proteins in sugar fed refractory mosquitoes without any blood feeding or immune challenge suggests that they might have some impact on refractory phenotype present naturally in environment. The alterations in the midgut proteomes of *An. culicifacies* refractory species B documented here emphasize that it may directly or indirectly linked to the parasite apoptosis mechanisms. Therefore, these identified differential expressed proteins that are involved in essential growth functions, namely invasion, survival, feeding and development in natural strain of refractory *An. culicifacies* mosquitoes may reflect the phenotypic characteristics of the mosquitoes and will improve our understandings towards blood meal digestion process, parasite vector interactions and proteomes of other vectors of human diseases for development of novel vector control strategies. Utilization of refractory or anti pathogen genes of these natural refractory mosquitoes that have no survival and fitness issues can be a promising strategy for genetic control of mosquito and emerge as a realistic prospect in future against malaria control.

## Methods

### Study design

**Fig. 8:**
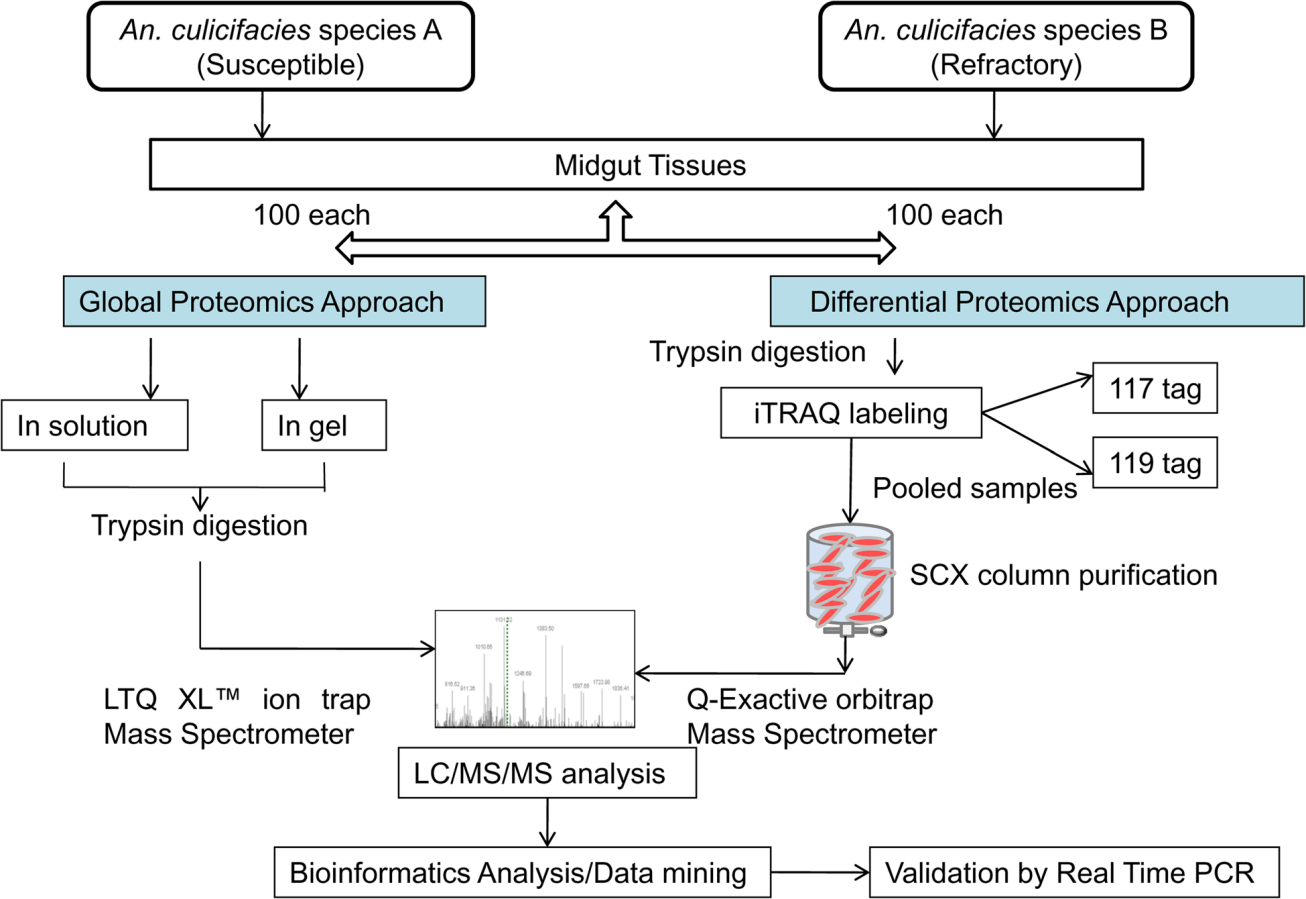
Schematic outline of the experimental workflow shown shot gun Proteomics and Differential Proteomics approach to map *An. culicifacies* species A (susceptible) and *An. culicifacies* species B (refractory) midguts and validation by Real time PCR

The present study was carried out on the midgut of sugar fed *An. culicifacies* species A (susceptible) and *An. culicifacies* species B (refractory). This study was designed in three steps; firstly, basic proteomic studies were carried out using in solution and Sodium dodecyl sulphate-polyacrylamide gel electrophoresis (SDS-PAGE)–in gel trypsin digestion approach followed by LC/MS/MS. Secondly, differential protein expression studies were carried out using iTRAQ labeling method. Finally, validation of few putative functional proteins was carried out using real time PCR (Fig. 8).

### Mosquitoes

Mosquitoes analyzed were 2–3-day old sugar fed *An. culicifacies* species A and *An. culicifacies* species B. These mosquitoes were reared and propagated in an insectary at National Institute of Malaria Research, Delhi under standard conditions as described by Adak et al. [42]. Adult mosquitoes were fed on water soaked raisins and 1% glucose solution. Establishment of refractory strain of *An. culicifacies* species B was previously described [4, 14]. This isofemale line has been identified and designated as *P. vivax* refractory strain *An. culicifacies* species B, originated from Haldwani, Uttaranchal State, India.

### Midgut collection and extract preparation

*An. culicifacies* species A and species B midgut (100) were dissected respectively and collected as a replicates. Briefly, midgut tissue was dissected in phosphate-buffered saline (PBS) buffer (0.15 M KH_2_PO_4_, 0.15 M Na_2_HPO_4_and 0.9% NaCl). Before sonication, lysis buffer (50 mM Tris HCL, 150 mM NaCl, 1% NP40) was added to the dissected tissue with protease inhibitor cocktail tablet (Complete, Roche Diagnostics, Germany). Midgut tissues were ultrasonicated in lysis buffer for 3 pulses of 20 s on ice and homogenized sample were centrifuged at 5000 rpm for 10 min at 4 °C. Debris was removed and supernatant was stored at − 20 °C. Total protein concentration was quantified by Bradford method (Sigma) using bovine serum albumin (BSA) as a standard. For RNA isolation experiments, dissected midguts were stored in RNA later at − 80 °C.

### In solution digestion

The extracted midgut lysates were reduced, alkylated and digested with trypsin after quantification. Briefly, 50 μg lysates were denatured with urea (4 M) and reduced with dithiothreitol (DTT,10 mM) for 1 h at 56 °C. Next step was the alkylation of reduced protein with iodoacetamide (IAA, 25 mM) for 30 min at 25 °C. Ammonium bicarbonate (100 mM) was further added in order to dilute urea to a final concentration of 0.5 M. Last step was the protein digestion in which trypsin was added to a protein mixture with concentration about 50 times lower than protein mixture and incubated it overnight at 37 °C. These tryptic peptide mixtures were further cleaned and desalted using C18 packed ziptips prior to mass spectrometry analysis. Desalted peptides were dried down using a speed vac and reconstituted in 2% acetonitrile with 0.1% Formic acid.

### In-gel digestion

In this approach, midgut extract was first resolved on 12% SDS-PAGE using a Bio-Rad apparatus (Bio-Rad, USA). Each band from the gel was excised and stored in separate tubes in 50 μl of stop solution (2% acetic acid) at − 20 °C for trypsin digestion. Each silver stained gel bands were destained, reduced, alkylated and digested with trypsin. A comprehensive method was described in our earlier publication [43]. Extracted peptides were dried down using a speed vac and reconstituted in 2% acetonitrile with 0.1% Formic acid.

### Mass spectrometry

LC/MS/MS analysis of the tryptic peptides obtained after in-solution and in-gel protein digests was carried out using LTQ XL™ ion trap mass spectrometer (Thermo Scientific). Separation was performed on PicoFrit C18 nanospray column (New Objective) of 360 um OD x 75um ID x 15um tip opening dimensions. Samples (15 μl each) were injected using a Thermo Scientific Surveyor Autosampler operated in the no waste injection mode with flow rate of 300 nl/min. Peptides were eluted from the in-solution digests at a linear acetonitrile gradient from 2 to 35% for over 210 min, and from the in-gel digests using a linear acetonitrile gradient from 2 to 32% for over 85 min, followed by high and low organic washes for another 5 min. The eluent is introduced directly to the LTQ XL mass spectrometer (Thermo Scientific) via a nanospray source with the spray voltage 1.8 kV and the ion transfer capillary set at 180 °C. MS data was acquired using a data-dependent top 7 method where a full MS scan from m/z 350–1500 was followed by MS/MS scans on the seven most intense ions. Each ion was subjected to Collision Induced Dissociation (CID) for fragmentation and peptide identification.

### Bioinformatics analysis

LC/MS/MS derived data were searched and analyzed using Proteome Discoverer 1.4 (Thermo Scientific). The acquired MS/MS data were searched against UniProt mosquito database, a well curated database using SEQUEST algorithm. Search parameters used: trypsin enzyme with up to two missed cleavages per peptide, precursor mass tolerance ±5000 ppm, fragment mass tolerance ±2 Da, Static modification: Carbamidomethyl Cysteine and variable modification: Oxidation of Methionine. Target Decoy peptide-spectrum match (PSM) Validator was used for PSM validation in database searches. The False discovery rate was set as Target FDR (Strict): 0.01 and Target FDR (Relaxed): 0.05. Peptide-level filters (high confidence peptides only) were used. Filter parameters were: Delta Cn =0.05, X-correlation scores = > than 1.5, 2.0, and 2.5 for two or more unique peptides and respective charge states of + 1, + 2, and + 3. Further molecular functions and biological process were identified using GO standards. Conserve domains and signal peptides were identified using SMART programme (http://smart.embl-heidelberg.de/) and Interproscan analysis. Cellular localization was depicted using CELLO (http://cello.life.nctu.edu.tw/).

### ITRAQ labeling and LC/MS/MS analysis

The midgut samples of both species A and species B of *An. culicifacies* were digested using trypsin and labeled with iTRAQ 8-plex reagent (tag 117- species A and 119- species B) using 8 plex Protein Quantitation kit (AB Sciex) according to manufacturer’s protocol. Labeled peptides were mixed and cleaned by strong cation exchange (SCX) chromatography and eluted from the SCX column using 250 mM, 350 mM and 450 mM ammonium acetate. Eluted peptides were dried and re-dissolved in 2% acetonitrile in 0.1% trifluoroacetic acid. Samples were loaded onto a 100-μm × 20 mm Magic C18 100 Å 5 U reverse phase trap where they were desalted online before being separated using a 75 μm × 150 mm Magic C18 200 Å 3 U reverse phase column. LC-MS/MS analysis was then carried out using a standard top 15 method on Thermo Scientific Q-Exactive orbitrap mass spectrometer in conjunction with a Proxeon Easy-nLC II HPLC (Thermo Scientific) and Proxeon nanospray source. MS/MS data was collected using data dependent mode and normalized higher energy collision dissociation (HCD) of 30 was used for fragmentation. MS1 automatic gain control (AGC) was set to 1e6 with an ion trap time of 60 ms, and MS2 AGC was set at 5e4 with 250 ms ion trap time. Unassigned charge states and charge states of + 1 and > + 6 were excluded for MS/MS selection. A dynamic exclusion of 15 s was set. Peptides were eluted using a flow rate of 300 nL/min and a gradient of 0.1% formic acid (A) and 100% acetonitrile (B). A 180-min gradient ran with 5 to 35% B over 155 min, 35 to 80% B over 10 min, 80% B for 2 min, 80 to 5% B over 3 min, and finally held at 5% B for 10 min. Each ion was subjected to CID for peptide identification followed by the Pulsed Q Dissociation (PQD) technique for iTRAQ quantitation.

### Database search and bioinformatics

The MS/MS spectra were searched and protein identification was performed using Proteome Discoverer 1.4 (Thermo Scientific). For MS/MS search both MASCOT and SEQUEST search engines were used against UniProt mosquito databases. Search parameters used were: trypsin enzyme (two missed cleavage); static modifications: Methylthio Cysteine, N-terminal iTRAQ 8-plex, and Lysine iTRAQ 8-plex; variable modification: oxidation of methionine; Precursor mass tolerance: 10 ppm, Fragment mass tolerance: 0.6 Da, Delta Correlation (Cn): 0.05. False discovery rate (FDR) was calculated using decoy database. FDR is set for 95% confidence for peptide ID’s. Peptide-level filters (high confidence peptides only) were used. Significant protein was validated on the basis of sequence coverage, peptide number and Cross correlation (Xcorr). Downregulated and upregulated proteins were identified using iTRAQ Ratio. Ratio > 1.5 is said to be up regulated, < 0.67 said to be down regulated and ratio from 1.5–0.67: Moderate to no change. Bioinformatics analysis of identified functional putative proteins were carried out using GO, SMART programme and CELLO. Network pathway for functional and protein interaction analyses as an evidence view was carried out using String 9.0 database (http://string-db.org/).

### Real time PCR

For the validation of iTRAQ method, RT-PCR was performed to detect the RNA level of the identified proteins. Briefly, RNA was isolated from midgut tissues using RNeasy micro kit (Qiagen) and reverse transcribed to cDNA using ReverTra Ace qPCR-RT kit (Toyobo) following the manufacturer’s instructions. RT-PCR assays were performed on Light Cycler 480 system (Roche Diagnostics, USA) using Thunderbird Sybr qPCR Mix (Toyobo). S7 RNA polymerase gene was used as a house keeping gene. PCR conditions were: Initial pre incubation (95 °C for 5 min) and denaturation-renaturation step of 40 cycles. Melting curve analysis was performed for further validation of amplification specificity. The comparative C_t_ method using the formula 2^- ΔΔCT^ method was employed to analyze the differential expression of respective genes. Following primers were designed and used: S7 [Fwd 5′cccaacaagcagaagagacc 3′ Rev. 5′cgactttgtgttcgatggtg 3′]; ADP/ATP: [Fwd 5′ggtatctctgccgctgtctc 3′ Rev. 5′ ggtcgggaagtaacggatca 3′]; SOD: [Fwd5’ tcaccaccagaagcatcaca 3’ Rev. 3’tttggaagtcacggttagc 5′]; Enolase: [Fwd5′ tgaaggcggttgagaacatc 3′ Rev. 5′ aacgaaacgcccagaatagc 3′].

Statistical analysis of real time PCR analyzed data were conducted with GraphPad Prism 5 software. Student’s t test was performed and *p* value (*p* < 0.05) was considered to be significant. The analysed data were given as a mean ± S.D.

## Additional files

Additional file 1: **Table S1.** A catalogue of midgut proteins identified using in-solution digestion strategy and LC/MS/MS in susceptible *An. culicifacies* species A. (DOCX 27 kb)

Additional file 2: **Table S2.** A catalogue of midgut proteins identified using in-solution digestion strategy and LC/MS/MS in in refractory *An. culicifacies* species B. (DOCX 27 kb)

Additional file 3: **Table S3.** A catalogue of midgut proteins identified using in-gel digestion strategy coupled with LC/MS/MS in *An. culicifacies* species A. (DOC 95 kb)

Additional file 4: **Table S4.** A catalogue of midgut proteins identified using in-gel digestion strategy coupled with LC/MS/MS in *An. culicifacies* species B. (DOC 97 kb)

Additional file 5: **Table S5.** A catalogue of identified putative proteins found in both species A and species B of *An. culicifacies* using iTRAQ labeling method. (DOCX 31 kb)

## Abbreviations

BSA: Bovine serum albumin
CID: Collision induced dissociation
Delta CN: Delta correlation
DTT: Dithiothreitol
FDR: False discovery rate
GO: Gene ontology
IAA: Iodoacetamide
iTRAQ: Isobaric tag for relative and absolute quantitation
KEGG: Kyoto encyclopedia of genes and genomes
LC/MS/MS: Liquid chromatography/mass spectrometry
NAD: Nicotinamide adenine dinucleotide
PBS: Phosphate-buffered saline
PCR: Polymerase chain reaction
PQD: Pulsed Q dissociation
ROS: Reactive oxygen species
SCX: Strong cation exchange
SDS-PAGE: Sodium dodecyl sulphate-polyacrylamide gel electrophoresis
SOD: Superoxide dismutase
WHO: World Health Organization
Xcorr: Cross correlation

## References

[1] World Malaria Report. WHO-World Health Organization. Geneva; 2016.

[2] Sharma VP (1999). Current scenario of malaria in India. Parassitologia.

[3] Subbarao SK, Adak T, Vasantha K, Joshi H, Raghavendra K, Cochrane AH, Nussenzweig RS, Sharma VP (1998). Susceptibility of *Anopheles culicifacies* species a and B to *Plasmodium vivax* and *Plasmodium falciparum* as determined by immunoradiometric assays. Trans R Soc Trop Med Hyg.

[4] Adak T, Singh OP, Nanda N, Sharma VP, Subbarao SK (2006). Isolation of a *Plasmodium vivax* refractory *Anopheles culicifacies* strain from India. Trop Med Int Health.

[5] Coluzzi M, Sabatini A, Petrarca V, Di Deco MA (1977). Behavioral divergences between mosquitoes with different inversion karyotypes in polymorphic populations of the *Anopheles gambiae* complex. Nature.

[6] Drexer AL, Vodovotz Y, Luckhart S (2008). *Plasmodium* development in the mosquito: biology bottlenecks and opportunities for mathematical modeling. Trends Parasitol.

[7] Whitten MMA, Shiao SH, Levashina EA (2006). Mosquito midguts and malaria: cell biology, compartmentalization and immunology. Parasite Immunol.

[8] Coleman J, Juhn J, James AA (2007). Dissection of midgut and salivary glands from ae. Aegypti mosquitoes. J Vis Exp.

[9] Han YS, Thompson J, Kafatos FC, Barillas-Mury C (2000). Molecular interactions between *Anopheles stephensi* midgut cells and *Plasmodium berghei*: the time bomb theory of ookinete invasion of mosquitoes. EMBO J.

[10] Vogel G (2010). The ‘do unto others’ malaria vaccine. Science.

[11] Vernick KD, Fujioka H, Seeley DC, Tandler B, Aikawa M, Miller LH (1995). Plasmodium gallinaceum: a refractory mechanism of ookinete killing in the mosquito, Anopheles gambiae. Exp Parasitol.

[12] Dimopoulos G (2003). Insect immunity and its implication in mosquito-malaria interactions. Cell Micro biol.

[13] Collins FH, Sakai RK, Vernick KD, Paskewitz S, Seeley DC, Miller LH, Collins WE, Campbell CC, Gwadz RW (1986). Genetic selection of a Plasmodium-refractory strain of the malaria vector *Anopheles gambiae*. Science.

[14] Vijay S, Rawat M, Adak T, Dixit R, Nanda N, Srivastav H, Sharma JK, Prasad GBKS, Sharma A (2011). Parasite killing in malaria non-vector mosquito *Anopheles culicifacies* species B: implication of nitric oxide synthase upregulation. PLoS One.

[15] Sharma A, Rodrigues J, Kajla MK, Agrawal N, Adak T, Bhatnagar RK (2010). Expression profile of Prophenoloxidase-encoding (*acppo6*) gene of *Plasmodium vivax*-refractory strain of *Anopheles culicifacies*. J Med Entomol.

[16] Rodrigues J (2007). Transcriptional analysis of an immune-responsive serine protease from Indian malarial vector, *Anopheles culicifacies*. BMC Mol Biol.

[17] Lefevre T, Vantaux A, Dabire KR, Mouline K, Cohuet A (2013). Non-genetic determinants of mosquito competence for malaria parasites. PLoS Pathog.

[18] Vargas L, Boyd M (1949). Culicine and aedine mosquitoes and the malaria infections of lower animals. Malariology.

[19] Menge DM, Daibin Z, Tom G, Louis G, John G, John B, Guiyun Y (2006). Quantitative trait loci controlling refractoriness to *Plasmodium falciparum* in natural *Anopheles gambiae* mosquitoes from a malaria-endemic region in western Kenya. Genetics.

[20] Habtewold T, Povelones M, Blagborough AM, Christophides GK (2008). Transmission blocking immunity in the malaria non-vector mosquito *Anopheles quadriannulatus* species a. PLoS Pathog.

[21] Rawal R, Vijay S, Kadian K, Singh J, Pande V, Sharma A (2016). Towards a proteomic catalogue and differential annotation of salivary gland proteins in blood fed malaria vector *Anopheles culicifacies* by mass spectrometry. PLoS One.

[22] Terra WR, Ferreira C (1994). Insect digestive enzymes: properties, compartmentalization and function. Comp Biochem Physiol B.

[23] Barillas-Mury C, Wells MA (1993). Cloning and sequencing of the bloodmeal-induced late trypsin gene from the mosquito *Aedes aegypti* and characterization of the upstream regulatory region. Insect Mol Biol.

[24] Borges-Veloso A, Saboia-Vahia L, Dias-Lopes G, Domont GB, Britto C, Cuervo P, De Jesus JB (2015). In-depth characterization of trypsin-like serine peptidases in the midgut of the sugar fed *Culex quinquefasciatus*. Parasit Vectors.

[25] Gorman MJ, Paskewitz SM (2001). Serine proteases as mediators of mosquito immune responses. Insect Biochem Mol Biol.

[26] Dias-Lopes G, Borges-Veloso A, Saboia-Vahia L, Domont GB, Britto C, Cuervo P, Jesus JBD (2015). Expression of active trypsin-like serine peptidases in the midgut of sugar-feeding female *Anopheles aquasalis*. Parasit Vectors..

[27] Danielli A, Kafatos FC, Loukeris TG (2003). Cloning and characterization of four *Anopheles gambiae* serpin isoforms, differentially induced in the midgut by *Plasmodium berghei* invasion. J Bio Chem.

[28] Goncalves RLS, Oliveira JHM, Oliveira GA, Andersen JF, Oliveira MF, Oliveira PL, Barillas-Mury C (2012). Mitochondrial reactive oxygen species modulate mosquito susceptibility to Plasmodium infection. PLoS One.

[29] Arsenijevic D, Onuma H, Pecqueur C, Raimbault S, Manning BS, Miroux B, Couplan E, Arsenijevic D, Alves-Guerra MC, Goubern M, Surwit R, Bouillaud F, Richard D, Collins S, Ricquier D (2000). Disruption of the uncoupling protein-2 gene in mice reveals a role in immunity and reactive oxygen species production. Nat Genet.

[30] Pebay-Peyroula E, Dahout-Gonzalez C, Kahn R, Trezeguet V, Lauquin GJ, Brandolin G (2003). Structure of mitochondrial ADP/ATP carrier in complex with carboxyatractyloside. Nature.

[31] Molina-Cruz A, DeJong RJ, Charles B, Gupta L, Kumar S, Jaramillo-Gutierrez G, Barillas-Mury C (2008). Reactive oxygen species modulate *Anopheles gambiae* immunity against bacteria and Plasmodium. J Biol Chem.

[32] Kumar S, Christophides GK, Cantera R, Charles B, Han YS, Meister S, Dimopoulos G, Kafatos FC, Barillas-Mury C (2003). The role of reactive oxygen species on *Plasmodium* melanotic encapsulation in *Anopheles gambiae*. Proc Natl Acad Sci U S A.

[33] Apte-Deshpande A, Paingankar M, Gokhale MD, Deobagkar DN (2012). *Serratia odorifera* a midgut inhabitant of *Aedes aegypti* Mosquito enhances its susceptibility to Dengue-2 virus. PLoS One.

[34] Kuadkitkan A, Wikan N, Fongsaran C, Smith DR (2010). Identification and characterization of prohibitin as a receptor protein mediating DENV-2 entry into insect cells. Virology.

[35] Chou WY, Huang SM, Liu YH, Chang GG (1994). Cloning and expression of pigeon liver cytosolic NADP+−dependent malic enzyme cDNA and some of its abortive mutants. Arch Biochem Biophys.

[36] Tchankouo-Nguetcheu S, Bourguet E, Lenormand P, Rousselle J-C, Namane A, Choumet V (2012). Infection by chikungunya virus modulates the expression of several proteins in *Aedes aegypti* salivary glands. Parasit Vectors..

[37] Zhou D, Visk DW, Haddad GG (2009). *Drosophila*, a golden bug, for the dissection of the genetic basis of tolerance and susceptibility to hypoxia. Pediatric Res.

[38] Starnes GL, Coincon M, Sygusch J, Sibley LD (2009). Aldolase is essential for energy production and bridging Adhesin-actin cytoskeletal interactions during parasite invasion of host cells. Cell Host Microbe.

[39] Rodriguez Mdel C, Martínez-Barnetche J, Alvarado-Delgado A, Batista C, Argotte-Ramos RS, Hernandez-Martínez S, Gonzalez Ceron L, Torres JA, Margos G, Rodríguez MH (2007). The surface protein Pvs25 of *Plasmodium vivax* ookinetes interacts with calreticulin on the midgut apical surface of the malaria vector *Anopheles albimanus*. Mol Biochem Parasitol.

[40] Dizaji NB, Basseri HR, Naddaf SR, Heidari M (2014). Molecular characterization of calreticulin from *Anopheles stephensi* midgut cells and functional assay of the recombinant calreticulin with *Plasmodium berghei* ookinetes. Gene.

[41] Atkinson SC, Armistead JS, Mathias DK, Sandeu MM, Tao D, Borhani-Dizaji N, Tarimo BB, Morlais I, Dinglasan RR, Borg N (2015). Structural analysis of *Anopheles* midgut aminopeptidase N reveals a novel malaria transmission-blocking vaccine B-cell epitope. Nat Struct Mol Biol.

[42] Adak T, Kaur S, Singh OP (1999). Comparative susceptibility of different members of the *Anopheles culicifacies* complex to *Plasmodium vivax*. Trans R Soc Trop Med Hyg.

[43] Vijay S, Rawat M, Sharma A (2014). Mass spectrometry based proteomic analysis of salivary glands of urban malaria vector *Anopheles stephensi*. Biomed Res Int.

