# Supplementary material for: Proteome-wide analysis of Anopheles culicifacies mosquito midgut: new insights into the mechanism of refractoriness
Source: BMC Genomics. 2018 May 8;19:337. doi: 10.1186/s12864-018-4729-3 (PMC5941458; doi:10.1186/s12864-018-4729-3)
Supplement: Supplementary file 1 — Table S1. A catalogue of midgut proteins identified using in-solution digestion strategy and LC/MS/MS in susceptible An. culicifacies species A. (DOCX 27 kb) [file 12864_2018_4729_MOESM1_ESM.docx]

Table S1 A catalogue of midgut proteins identified using in-solution digestion strategy and LC/MS/MS in susceptible *An. culicifacies* species A

| **S.no** | **Accession number*** | **Protein** | **Sequence Coverage** | **Peptides** | **M.wt (kDa)** | **C. pI** | **Function** |
| --- | --- | --- | --- | --- | --- | --- | --- |
| **CYTOSKELTAL PROTEINS** | | | | | | | |
| 1. 1 | GI:158299190 | AGAP010147-PA (Similar to *An. gambiae*) | 24 | 48 | 224.2 | 5.76 | ATP binding/motor activity |
|  | GI:94468486 | Actin ( Similar to *Ae. aegypti*) | 42 | 9 | 41.8 | 5.48 | ATP binding |
| 1. 17 | GI:158289879 | AGAP010435-PA (similar to *An. gambiae)* | 3 | 5 | 513 | 5.55 | [microtubule motor activity](http://www.ebi.ac.uk/QuickGO/GTerm?id=GO:0003777)/ATP binding |
|  | GI:170033532 | Beta-1 tubulin (similar to *Aedes aegypti*) | 31 | 9 | 48.1 | 4.78 | [GTPase activity](http://www.ebi.ac.uk/QuickGO/GTerm?id=GO:0003924) cytoskelton |
|  | GI:1187918307 | AGAP009200-PA(similar to *Anopheles gambiae*) | 7 | 3 | 101 | 8.29 | [extracellular matrix structural constituent](http://www.ebi.ac.uk/QuickGO/GTerm?id=GO:0005201) |
| **ENERGY PRODUCTION** | | | | | | | |
|  | GI:568252760 | ATP synthase subunit beta (Similar to *An. darlingi*) | 57 | 21 | 53.7 | 5.12 | ATP binding, Ion transport |
|  | GI:347968691 | AGAP002858-PC  (similar to  *An. gambiae*) | 27 | 18 | 100 | 5.54 | [sodium: potassium-exchanging ATPase activity](http://www.ebi.ac.uk/QuickGO/GTerm?id=GO:0005391) |
|  | GI:31207753 | V-type proton ATPase catalytic subunit (similar to *Anopheles gambiae*) | 16 | 7 | 68.2 | 5.39 | [ATP binding](http://www.ebi.ac.uk/QuickGO/GTerm?id=GO:0005524), [proton-transporting ATPase activity, rotational mechanism](http://www.ebi.ac.uk/QuickGO/GTerm?id=GO:0046961) |
|  | GI:170048704 | vacuolar ATP synthase subunit ac39 (similar to *Culex quinquefasciatus)* | 14 | 3 | 39.6 | 4.94 | [hydrogen ion transmembrane transporter activity](http://www.ebi.ac.uk/QuickGO/GTerm?id=GO:0015078) |
| **TRANSPORT** | | | | | | | |
|  | GI:58391886 | AGAP009833-PA (similar to *An. gambiae)* | 29 | 6 | 30.7 | 8.56 | [voltage-gated anion channel activity](http://www.ebi.ac.uk/QuickGO/GTerm?id=GO:0008308), ion transport |
|  | GI:92090585 | ADP,ATP carrier protein (similar to *An. gambiae)* | 13 | 4 | 32.8 | 9.67 | Transport activity |
|  | GI:568258499 | Putative cytochrome c1 (similar to *Anopheles darlingi)* | 30 | 5 | 33.3 | 8.54 | Electron transport, iron binding |
|  | GI:158294869 | AGAP005845-PA (similar to *Anopheles gambiae*) | 10 | 3 | 44 | 7.75 | Transmembrane transport activity |
|  | GI:119113312 | AGAP011131-PA (similar to *Anopheles gambiae*) | 29 | 2 | 19.5 | 5.95 | [hydrogen ion transmembrane transporter activity](http://www.ebi.ac.uk/QuickGO/GTerm?id=GO:0015078) |
| **IMMUNE RELATED** | | | | | | | |
|  | GI:158297660 | AGAP011453-PA (similar to *An. gambiae)* | 10 | 5 | 85.8 | 5.14 | Iron ion transport |
|  | GI:158300735 | AGAP011938-PA (similar to *Anopheles gambiae*) | 11 | 5 | 68.1 | 7.18 | [structural molecule activity](http://www.ebi.ac.uk/QuickGO/GTerm?id=GO:0005198), role in transfer of lipid |
| **SIGNAL TRANSDUCTION** | | | | | | | |
|  | GI:170038163 | Putative uncharacterized protein (similar to *Culex quinquefasciatus*) | 13 | 2 | 32.3 | 5.59 | GTPase activator activity, signal transduction |
|  | GI:347966284 | AGAP001633-PA(similar to *Anopheles gambiae*) | 3 | 13 | 913.6 | 6.61 | protein serine/threonine kinase activity |
| **BINDING PROTEINS** | | | | | | | |
|  | GI:347970642 | Annexin (similar to *An. gambiae)* | 32 | 7 | 35.9 | 4.78 | [calcium-dependent phospholipid binding](http://www.ebi.ac.uk/QuickGO/GTerm?id=GO:0005544) |
|  | GI:445068730 | LANB2 (similar to *Anopheles merus*) | 4 | 3 | 179.3 | 5.72 | Mediate cell adhesion, growth migration, and differentiation |
|  | GI:170046970 | Ro ribonucleoprotein autoantigen (similar to *Culex quinquefasciatus*) | 7 | 2 | 68.4 | 9.31 | [RNA binding](http://www.ebi.ac.uk/QuickGO/GTerm?id=GO:0003723) |
|  | GI:118792103 | AGAP012401-PA (Similar to *An. gambiae*) | 16 | 8 | 57.2 | 5.74 | Catalytic activity, cation binding |
|  | GI:157126791 | Acyl carrier protein (similar to *Aedes aegypti)* | 16 | 2 | 17.8 | 5.36 | [fatty acid biosynthetic process](http://www.ebi.ac.uk/QuickGO/GTerm?id=GO:0006633) |
|  | GI:28569878 | Gag-like protein (similar to *An. gambiae)* | 8 | 2 | 59.5 | 9.88 | Nucleic acid binding, zinc binding |
|  | GI:158284488 | AGAP012757-PA (similar to *Anopheles gambiae*) | 6 | 3 | 76.1 | 5.12 | [zinc ion binding](http://www.ebi.ac.uk/QuickGO/GTerm?id=GO:0008270), [metallopeptidase activity](http://www.ebi.ac.uk/QuickGO/GTerm?id=GO:0008237) |
|  | GI:170034793 | Putative uncharacterized protein (similar to *Culex quinquefasciatus*) | 33 | 2 | 119 | 7.71 | protein binding |
|  | GI:118794438 | AGAP001622-PA(similar to *An. gambiae*) | 20 | 3 | 22.9 | 4.78 | [calcium ion binding](http://www.ebi.ac.uk/QuickGO/GTerm?id=GO:0005509) |
|  | GI:158287025 | AGAP005293-PB (similar to *Anopheles gambiae*) | 24 | 8 | 50.7 | 4.86 | [GTPase activity](http://www.ebi.ac.uk/QuickGO/GTerm?id=GO:0003924), [GTP binding](http://www.ebi.ac.uk/QuickGO/GTerm?id=GO:0005525) |
|  | GI:347964032 | AGAP000550-PA (similar to *An. gambiae)* | 4 | 6 | 164 | 5.52 | Cell matrix adhesion |
| **GLYCOLYSIS** | | | | | | | |
|  | GI:158292170 | AGAP004437-PA(similar to *Anopheles gambiae*) | 6 | 4 | 81.2 | 7.80 | [glycerol-3-phosphate dehydrogenase activity](http://www.ebi.ac.uk/QuickGO/GTerm?id=GO:0004368), [calcium ion binding](http://www.ebi.ac.uk/QuickGO/GTerm?id=GO:0005509) |
| **REDOX MECHANISM** | | | | | | | |
|  | GI:347966240 | AGAP001613-PA (similar to *Anopheles gambiae*) | 17 | 4 | 38.9 | 4.65 | cell redox homeostasis |
|  | GI:356578761 | Catalase (similar to *Anopheles aquasalis*) | 22 | 6 | 56.7 | 8.32 | [metal ion binding](http://www.ebi.ac.uk/QuickGO/GTerm?id=GO:0046872), [catalase activity](http://www.ebi.ac.uk/QuickGO/GTerm?id=GO:0004096) |
|  | GI:158300147 | AGAP012407-PA (Fragment) (similar to *Anopheles gambiae*) | 11 | 3 | 53.1 | 5.1 | [protein disulfide oxidoreductase activity](http://www.ebi.ac.uk/QuickGO/GTerm?id=GO:0015035), protein folding |
|  | GI:170068344 | NADH dehydrogenase iron-sulfur protein 8 (similar to *Culex quinquefasciatus*) | 25 | 4 | 24.3 | 5.67 | [oxidoreductase activity](http://www.ebi.ac.uk/QuickGO/GTerm?id=GO:0016651) |
|  | GI:347967370 | AGAP002197-PA (similar to *Anopheles gambiae*) | 13 | 2 | 41.9 | 7.52 | [iron ion binding](http://www.ebi.ac.uk/QuickGO/GTerm?id=GO:0005506), [oxidoreductase activity](http://www.ebi.ac.uk/QuickGO/GTerm?id=GO:0016705) |
| **MOLECULAR PROCESSES** | | | | | | | |
|  | GI:568251820 | Elongation factor 1-alpha (Fragment) (similar *to Anopheles darlingi)* | 16 | 5 | 49.6 | 9.03 | [GTPase activity](http://www.ebi.ac.uk/QuickGO/GTerm?id=GO:0003924), [translation elongation factor activity](http://www.ebi.ac.uk/QuickGO/GTerm?id=GO:0003746) |
|  | GI:170074058 | Histone H4 (similar to *Culex quinquefasciatus)* | 22 | 2 | 10.2 | 11.5 | DNA binding |
|  | GI:170048542 | Eukaryotic translation initiation factor 4 gamma 3 (similar to *Culex quinquefasciatus*) | 4 | 2 | 110.3 | 7.36 | [translation initiation factor activity](http://www.ebi.ac.uk/QuickGO/GTerm?id=GO:0003743), protein binding |
|  | GI:170039780 | Huntingtin interacting protein (similar to *Culex quinquefasciatus)* | 5 | 6 | 265 | 6.16 | [DNA binding](http://www.ebi.ac.uk/QuickGO/GTerm?id=GO:0003677) |
|  | GI:170049962 | Putative uncharacterized protein (similar to *Culex quinquefasciatus*) | 4 | 2 | 88 | 9.1 | histone acetyltransferase activity. |
| **UNKNOWN** | | | | | | | |
|  | T1E870 | Putative pdsw (similar to *An. aquasalis*) | 22 | 3 | 19.6 | 6.79 | unknown |
|  | GI:114864609 | Conserved protein (similar to *Anopheles funestus*) | 37 | 3 | 10 | 9.52 | Not known |
|  | GI:347966622 | AGAP001799-PB(similar to *Anopheles gambiae*) | 11 | 4 | 32.4 | 4.86 | Unknown |
|  | GI:158289831 | AGAP010479-PA (similar to *Anopheles gambiae*) | 8 | 3 | 45 | 6.8 | Unknown |
| **PROTEIN FOLDING/DIMERIZATION** | | | | | | | |
|  | GI:347963871 | AGAP000462-PA (similar to *An. gambiae*) | 25 | 3 | 18 | 8.48 | [protein folding](http://www.ebi.ac.uk/QuickGO/GTerm?id=GO:0006457), peptidyl-prolyl cis-trans isomerase activity |
|  | GI:373880220 | Calreticulin (similar to *An. stephensi*) | 23 | 6 | 46.2 | 4.54 | Protein folding, calcium ion binding |
|  | GI:170034322 | Bhlhzip transcription factor max/bigmax (similar to *Culex quinquefasciatus*) | 22 | 2 | 16.4 | 9.26 | protein dimerization activity |
| **MITOCHONDRION PROTEIN** | | | | | | | |
|  | GI:119113263 | AGAP011159-PA(similar to *An. gambiae)* | 21 | 3 | 17.3 | 5.41 | Cytochrome c oxidase activity |
|  | GI:157109957 | AAEL005435-PA (similar to Aedes aegypti) | 6 | 2 | 52.2 | 6.27 | [metalloendopeptidase activity](http://www.ebi.ac.uk/QuickGO/GTerm?id=GO:0004222) |
|  | GI:158291084 | AGAP002364-PA (similar to *Anopheles gambiae*) | 7 | 4 | 82 | 8.92 | Mitochondrion protein |
|  | GI:347966322 | AGAP001653-PA (similar to *Anopheles gambiae*) | 7 | 3 | 79.1 | 6.87 | [electron carrier activity](http://www.ebi.ac.uk/QuickGO/GTerm?id=GO:0009055), [NADH dehydrogenase (ubiquinone) activity](http://www.ebi.ac.uk/QuickGO/GTerm?id=GO:0008137) |
| **CATABOLIC PROCESS** | | | | | | | |
|  | GI:158297628 | AGAP011476  (similar to *An. gambiae)* | 8 | 6 | 101 | 5.68 | [Peptidoglycan catabolic process](http://www.ebi.ac.uk/QuickGO/GTerm?id=GO:0009253) |
