# Supplementary material for: Proteome-wide analysis of Anopheles culicifacies mosquito midgut: new insights into the mechanism of refractoriness
Source: BMC Genomics. 2018 May 8;19:337. doi: 10.1186/s12864-018-4729-3 (PMC5941458; doi:10.1186/s12864-018-4729-3)
Supplement: Supplementary file 2 — Table S2. A catalogue of midgut proteins identified using in-solution digestion strategy and LC/MS/MS in in refractory An. culicifacies species B. (DOCX 27 kb) [file 12864_2018_4729_MOESM2_ESM.docx]

Table S2 A catalogue of midgut proteins identified using in-solution digestion strategy and LC/MS/MS in refractory *An. culicifacies* species B

| **S.No** | **Accession number*** | **Protein/Similarity with species** | **Sequence Coverage** | **Peptides** | **M.wt (kDa)** | **Function** |
| --- | --- | --- | --- | --- | --- | --- |
| **CYTOSKELTAL PROTEINS** | | | | | | |
|  | GI:2654602 | chitinase (*Anopheles gambiae*) | 8 | 3 | 57.2 | Chitinase activity |
|  | GI:604775631 | putative collagen type iv, partial (*Aedes albopictus*) | 18 | 3 | 28.6 | formation of connective tissue structure |
|  | GI:158293004 | AGAP004877-PA (*An. gambiae*) | 7 | 4 | 102.8 | motor activity |
|  | GI:158299970 | AGAP009201-PA, partial (*An. gambiae*) | 3 | 2 | 93.4 | [Extracellular matrix structural constituent](http://www.ebi.ac.uk/QuickGO/GTerm?id=GO:0005201) |
|  | GI:158299190 | AGAP010147-PA (*An. gambiae*) | 17 | 27 | 224.2 | ATP binding/motor activity |
| **IMMUNE RELATED** | | | | | | |
|  | GI:410327 | Trypsinogen precursor of ANTRYP7 (*An. gambiae*) | 10 | 2 | 28.4 | Serine-type endopeptidase activity |
|  | GI:118784691 | AGAP004571-PA (*An. gambiae*) | 10 | 2 | 35.9 | serine-type endopeptidase activity |
|  | GI:668454383 | trypsin (*An. sinensis*) | 9 | 2 | 28.4 | serine-type endopeptidase activity |
|  | GI:1644281 | serine protease (*An. gambiae*) | 4 | 2 | 29.2 | serine-type endopeptidase activity |
|  | GI:240270402 | serpin 10 plasmodium-related inhibitory serine protease inhibitor, partial (*An. gambiae*) | 12 | 2 | 24.5 | Proteinase inhibitors |
|  | GI:474027 | chymotrypsinogen-like protease ANCHYM2 (*An. gambiae*) | 10.8 | 2 | 27.9 | [serine-type endopeptidase activity](http://www.ebi.ac.uk/QuickGO/GTerm?id=GO:0004252) |
| **RECEPTOR** | | | | | | |
|  | GI:668465571 | aminopeptidase N (*An. sinensis*) | 2 | 2 | 215.1 | metallopeptidase activity |
|  | GI:347969212 | AGAP003077-PA (*An. gambiae*) | 2 | 2 | 112.1 | metallopeptidase activity /ion binding |
|  | GI:158284488 | Anopheles gambiae str. PEST AGAP012757-PA, partial (*An. gambiae*) | 3 | 2 | 76.1 | metallopeptidase activity /ion binding |
|  | GI:158295810 | AGAP006400-PA (*An. gambiae*) | 5 | 2 | 56.4 | phosphatase activity |
|  | GI:347970794 | AGAP003869-PA (*An. gambiae*) | 8 | 3 | 54.0 | aminopeptidase activity |
|  | GI:158297815 | AGAP004809-PA (*An. gambiae*) | 5 | 4 | 113.2 | Metallopeptidase activity |
|  | GI:347970416 | AGAP013393-PA (*An. gambiae*) | 5 | 4 | 104.1 | [metallo aminopeptidase activity](http://www.ebi.ac.uk/QuickGO/GTerm?id=GO:0070006) |
| **BINDING PROTEINS** | | | | | | |
|  | GI:184191216 | maltase-like alpha-1,4-glucosidase, partial (*Aedes aegypti)* | 22 | 1 | 9.7 | Cation binding/Catalytic activity |
|  | GI:118792103 | AGAP012401-PA (*An. gambiae*) | 13 | 5 | 57.2 | Cation binding/Catalytic activity |
|  | GI:108878534 | AAEL005733-PA (*Aedes aegypti*) | 7 | 11 | 222.1 | ATP binding |
|  | GI:347970325 | AGAP003656-PB (*An. gambiae* str. PEST) | 2 | 4 | 450.9 | Protein binding |
|  | GI:158286589 | AGAP006936-PB (*An. gambiae*) | 11 | 2 | 32.7 | electron carrier activity, heme binding |
|  | GI:118789564 | AGAP007963-PA (*An. gambiae*) | 17 | 2 | 22.1 | calcium ion binding |
|  | GI:668445830 | AGAP007120-PA-like protein (*An. sinensis*) | 19 | 2 | 17.0 | ATP binding |
|  | GI:347964032 | AGAP000550-PA (*An. gambiae)* | 5 | 6 | 164 | Cell matrix adhesion |
|  | GI:668462689 | hypothetical protein ZHAS_00018220 (*An. sinensis*) | 4 | 3 | 121.9 | cation binding/Catalytic activity |
|  | GI:158284413 | AGAP012930-PA, partial (*An. gambiae)* | 45 | 4 | 12.1 | [calcium-dependent phospholipid binding](http://www.ebi.ac.uk/QuickGO/GTerm?id=GO:0005544), |
|  | GI:167865824 | calmodulin (*Culex quinquefasciatus*) | 54.5 | 3 | 7.5 | [calcium ion binding](http://www.ebi.ac.uk/QuickGO/GTerm?id=GO:0005509) |
|  | GI:158300570 | AGAP012071-PA (*An. gambiae*) | 15 | 2 | 20.5 | [lactoylglutathione lyase activity](http://www.ebi.ac.uk/QuickGO/GTerm?id=GO:0004462)/ [metal ion binding](http://www.ebi.ac.uk/QuickGO/GTerm?id=GO:0046872) |
|  | GI:58380021 | AGAP003721-PA (*An. gambiae*) | 9 | 2 | 35.8 | [calcium-dependent phospholipid binding](http://www.ebi.ac.uk/QuickGO/GTerm?id=GO:0005544) |
| **GLYCOLYSIS** | | | | | | |
|  | GI:347966276 | AGAP001630-PA (similar to *An. gambiae*) | 35 | 6 | 26.3 | Triose-phosphate isomerase activity |
|  | GI:158287395 | AGAP011208-PA, partial (*An. gambiae*) | 13 | 2 | 27.3 | [ATP binding](http://www.ebi.ac.uk/QuickGO/GTerm?id=GO:0005524)/ [fructokinase activity](http://www.ebi.ac.uk/QuickGO/GTerm?id=GO:0008865) |
| **REDOX MECHANISM** | | | | | | |
|  | GI:40792585 | catalase 1, partial (*An. gambiae*) | 13 | 3 | 54.7 | Heme binding , catalase activity |
|  | GI:668463170 | hypothetical protein ZHAS_00018905 (*An. sinensis*) | 2 | 2 | 269.9 | oxidoreductase activity |
|  | GI:158296454 | AGAP000881-PA (*An. gambiae*) | 14 | 5 | 58.3 | oxidoreductase activity |
|  | GI:668453175 | AGAP000909-PA-like protein (An. sinensis) | 9.6 | 3 | 46.6 | [cell redox homeostasis](http://www.ebi.ac.uk/QuickGO/GTerm?id=GO:0045454) |
|  | GI:668465162 | AGAP004366-PA-like protein (*An. sinensis*) | 6 | 2 | 63.0 | [oxidoreductase activity](http://www.ebi.ac.uk/QuickGO/GTerm?id=GO:0016620) |
|  | GI:472232649 | glyceraldehyde-3-phosphate dehydrogenase, partial (*An. stephensi*) | 21 | 2 | 19.1 | oxidoreductase activity |
| **TRANSPORT** | | | | | | |
|  | GI:58391886 | AGAP009833-PA (*An. gambiae* ) | 21 | 4 | 30.7 | [Voltage-gated anion channel activity](http://www.ebi.ac.uk/QuickGO/GTerm?id=GO:0008308)/Protein transport |
|  | GI:1438862 | ADP/ATP carrier protein (*An. gambiae*) | 9 | 2 | 32.8 | Energy transfer |
|  | GI:668447909 | AGAP007790-PA-like protein (*An. sinensis*) | 11 | 2 | 36.2 | [potassium ion transport](http://www.ebi.ac.uk/QuickGO/GTerm?id=GO:0006813) |
|  | GI:167875207 | PfmpC (*Culex quinquefasciatus*) | 12 | 4 | 38.4 | [transport](http://www.ebi.ac.uk/QuickGO/GTerm?id=GO:0006810) |
|  | GI:158302485 | AGAP001138-PA (*An. gambiae*) | 14.8 | 2 | 26.9 | [ATP synthesis coupled proton transport](http://www.ebi.ac.uk/QuickGO/GTerm?id=GO:0015986) |
|  | GI: 28193044 | cytochrome oxidase subunit II, partial (mitochondrion) (*An. culicifacies C*) | 26 | 3 | 20.2 | [cytochrome-c oxidase activity](http://www.ebi.ac.uk/QuickGO/GTerm?id=GO:0004129) |
|  | GI: 604777549 | putative mitochondrial phosphate carrier protein (*Aedes albopictus)* | 11 | 4 | 37.7 | [transport](http://www.ebi.ac.uk/QuickGO/GTerm?id=GO:0006810) |
|  | GI:38196217 | transferrin-like, partial [Anopheles gambiae] | 18 | 2 | 21.4 | Ion transport |
|  | GI:99082886 | putative cytoplasmic carbonic anhydrase (*An. gambiae*) | 12 | 2 | 31.4 | Ion transport and pH regulation |
| **CELL DIFFERENTIATION** | | | | | | |
|  | GI:668445830 | AGAP007120-PA-like protein (*An. sinensis*) | 19 | 2 | 17.0 | ATP binding |
| **HYDROLASE / CATALYTIC ACTIVITY** | | | | | | |
|  | GI:668457401 | AGAP003995 (*An. gambiae*) | 5 | 2 | 72 | Hydrolase activity |
|  | GI:158300733 | AGAP011939-PA, partial (*An. gambiae*) | 14 | 3 | 35.9 | [catalytic activity](http://www.ebi.ac.uk/QuickGO/GTerm?id=GO:0003824) |
|  | GI:58386478 | AGAP008684-PA (*An. gambiae*) | 8 | 3 | 52.0 | [cis-stilbene-oxide hydrolase activity](http://www.ebi.ac.uk/QuickGO/GTerm?id=GO:0033961) |
|  | GI:158299096 | AGAP010056-PA (*An. gambiae*) | 9 | 3 | 63.3 | [beta-N-acetylhexosaminidase activity](http://www.ebi.ac.uk/QuickGO/GTerm?id=GO:0004563) |
| **UNKNOWN** | | | | | | |
|  | GI:114864609 | conserved protein (*An. funestus*) | 36 | 2 | 10.0 | Unknown |
|  | GI:158289831 | AGAP010479-PA (*An. gambiae)* | 5.6 | 2 | 45.0 | Unknown |
|  | GI:108872537 | AAEL011180-PA (*Aedes aegypti*) | 7.5 | 2 | 30.9 | Unknown |
|  | GI:158293034 | AGAP004860-PA (*An. gambiae*) | 5 | 4 | 104.1 | Unknown |
|  | GI:347973231 | AGAP010132-PA PA (*An. gambiae*) | 6 | 2 | 55.8 | Unknown |
|  | GI:668452383 | AGAP003656-PB-like protein (*An. sinensis*) | 2 | 3 | 168.3 | Unknown |
| **PROTEIN FOLDING** | | | | | | |
|  | GI:347963873 | AGAP000462-PB (*An. gambiae*) | 22 | 2 | 18.0 | [peptidyl-prolyl cis-trans isomerase activity](http://www.ebi.ac.uk/QuickGO/GTerm?id=GO:0003755) |
|  | GI:343197955 | Calreticulin (*An. stephensi*) | 7 | 2 | 46.2 | Protein folding, calcium ion binding |
| **ENERGY PRODUCTION/RESERVE** | | | | | | |
|  | GI:158290453 | AGAP002858-PA (*An. gambiae*) | 5 | 3 | 110.9 | [sodium: potassium-exchanging ATPase activity](http://www.ebi.ac.uk/QuickGO/GTerm?id=GO:0005391) |
|  | GI:158297275 | AGAP007939-PA (An. gambiae)] | 4 | 3 | 96.8 | [glycogen phosphorylase activity](http://www.ebi.ac.uk/QuickGO/GTerm?id=GO:0008184) |
| **MOLECULAR PROCESS** | | | | | | |
|  | GI:604779658 | putative elongation factor 1 alpha, partial [Aedes albopictus] | 9 | 2 | 49.6 | [GTPase activity](http://www.ebi.ac.uk/QuickGO/GTerm?id=GO:0003924)/ [GTP binding](http://www.ebi.ac.uk/QuickGO/GTerm?id=GO:0005525) [translation elongation factor activity](http://www.ebi.ac.uk/QuickGO/GTerm?id=GO:0003746) |
|  | GI:108880805 | AAEL015683-PA (*Aedes aegypti)* | 20 | 2 | 13.6 | DNA binding/ [protein heterodimerization activity](http://www.ebi.ac.uk/QuickGO/GTerm?id=GO:0046982) |
|  | GI:604773915 | putative ubiquitin a-52 residue ribosomal protein fusion product 1, partial (*Aedes albopictus*) | 46 | 3 | 24.7 | protein binding/  Regulation |
