# Supplementary material for: Proteome-wide analysis of Anopheles culicifacies mosquito midgut: new insights into the mechanism of refractoriness
Source: BMC Genomics. 2018 May 8;19:337. doi: 10.1186/s12864-018-4729-3 (PMC5941458; doi:10.1186/s12864-018-4729-3)
Supplement: Supplementary file 3 — Table S3. A catalogue of midgut proteins identified using in-gel digestion strategy coupled with LC/MS/MS in An. culicifacies species A. (DOC 95 kb) [file 12864_2018_4729_MOESM3_ESM.doc]

Table S3 A catalogue of midgut proteins identified using in-gel digestion strategy coupled with LC/MS/MS in *An. culicifacies* species A

| **S.no** | **Accession number** | **Protein** | **Sequence Coverage** | **M.wt (kDa)** | **C. pI** | **Function** |
| --- | --- | --- | --- | --- | --- | --- |
| **BAND 1** | | | | | | |
| 1. **1** | GI:668461709 | myosin heavy chain, isoform N (similar to *Anopheles sinensis*) | 2 | 215.2 | 6.30 | Motor activity /ATP binding |
| 1. **2.** | GI:59709763 | actin, partial ( similar to *Anopheles stephensi*) | 18 | 18.9 | 5.77 | Cell motility |
|  | GI:15082050 | sodium/potassium transporting ATPase alpha chain, partial (similar to *Aedes aegypti)* | 8 | 22.9 | 6.54 | Nucleotide binding |
| 1. 1 | GI:356578765 | Superoxide dismutase [Cu-Zn] (similar to *Anopheles aquasalis*) | 7 | 36.9 | 9.60 | [metal ion binding](http://www.ebi.ac.uk/QuickGO/GTerm?id=GO:0046872) |
| 1. 2 | GI:157133304 | AAEL012710-PB (similar to *Aedes aegypti*) | 5 | 16.6 | 8.43 | Signal Transduction |
| **BAND 3** | | | | | | |
|  | **GI:347967370** | **AGAP002197-PA (similar to *Anopheles gambiae*)** | **7** | **41.9** | **7.52** | **iron ion binding/**[**mono oxygenase activity**](http://www.ebi.ac.uk/QuickGO/GTerm?id=GO:0004497) |
|  | GI:158300040 | AGAP009257-PA (similar to *Anopheles gambiae*) | 4 | 33.7 | 7.87 | Inactivation of receptors |
|  | GI:157130044 | AAEL000308-PA (similar to *Aedes aegypti*) | 6 | 18.2 | 9.54 | cell adhesion ,/tissue regeneration |
| **BAND 4** | | | | | | |
|  | **GI:158298265** | **AGAP003995-PA (similar to *Anopheles gambiae*)** | **4** | **73** | **5.31** | **hydrolase activity** |
|  | GI:170054451 | General odorant-binding protein 56d (similar to *Culex quinquefasciatus)* | 9 | 14 | 8.43 | [odorant binding](http://www.ebi.ac.uk/QuickGO/GTerm?id=GO:0005549) |
| **BAND 6** | | | | | | |
|  | GI:568251743 | Alkaline phosphatase (Fragment) (similar to *Anopheles darlingi*) | 3 | 35 | 7.27 | [alkaline phosphatase activity](http://www.ebi.ac.uk/QuickGO/GTerm?id=GO:0004035) |
| **BAND 8** | | | | | | |
|  | **GI:**  **118792103** | **AGAP012401-PA (similar to *Anopheles gambiae*)** | **17** | **57.2** | **5.74** | **Catalytic activity, ion binding** |
|  | GI:167865603 | citron ser/thr kinase (similar to *Culex quinquefasciatus*) | 4 | 68.2 | 8.76 | protein kinase activity /ATP binding |
| **BAND 9** | | | | | | |
|  | GI:157111313 | AAEL015297-PA (similar to *Aedes aegypti*) | 2 | 87.4 | 8.25 | DNA binding |
|  | GI:158285361 | AGAP007604-PA (similar to *Anopheles gambiae*) | 3 | 48 | 8.06 | [transferase activity](http://www.ebi.ac.uk/QuickGO/GTerm?id=GO:0016757) |
|  | GI:170051650 | Rho-associated protein kinase 1 (similar to *Culex quinquefasciatus)* | 2 | 160 | 6.44 | [serine/threonine kinase activity](http://www.ebi.ac.uk/QuickGO/GTerm?id=GO:0004674), [ATP binding](http://www.ebi.ac.uk/QuickGO/GTerm?id=GO:0005524) |
|  | GI:170029564 | Juvenile hormone epoxide hydrolase (similar to *Culex quinquefasciatus)* | 6 | 51.7 | 8.41 | [hydrolase activity](http://www.ebi.ac.uk/QuickGO/GTerm?id=GO:0033961) |
|  | GI:157117978 | AAEL008128-PA (similar to *Aedes aegypti*) | 21 | 10.2 | 7.74 | [metal ion binding](http://www.ebi.ac.uk/QuickGO/GTerm?id=GO:0046872) |
|  | GI:170055993 | FKBP-rapamycin associated protein (similar to *Culex quinquefasciatus)* | 19 | 11.3 | 11.28 | Unknown |
|  | T1DG66 | Ubiquitin carboxyl terminal hydrolase (similar to *Anopheles aquasalis)* | 5 | 25.3 | 5.07 | [protease activity](http://www.ebi.ac.uk/QuickGO/GTerm?id=GO:0004843) |
|  | GI:66863200 | Putative reverse transcriptase (Fragment) (similar to *Anopheles stephensi*) | 5 | 13.3 | 9.91 | [RNA binding](http://www.ebi.ac.uk/QuickGO/GTerm?id=GO:0003723), [DNA polymerase activity](http://www.ebi.ac.uk/QuickGO/GTerm?id=GO:0003964) |
| **BAND 10** | | | | | | |
|  | GI:170042346 | DCAPL2 (similar to *Culex quinquefasciatus)* | 2 | 169 | 9.76 | Unknown |
|  | GI:157131250 | AAEL012054  (similar to *Aedes aegypti*) | 2 | 74 | 6.83 | [thiol oxidase activity](http://www.ebi.ac.uk/QuickGO/GTerm?id=GO:0016972) |
| **BAND 11** | | | | | | |
|  | GI:355398645 | ATP synthase beta subunit (Fragment) (similar to *Aedes albopictus*) | 14 | 17.3 | 4.93 | [ATP binding](http://www.ebi.ac.uk/QuickGO/GTerm?id=GO:0005524) |
|  | **GI:158299190** | **AGAP010147-PA (similar *to Anopheles gambiae)*** | **2** | **224** | **5.76** | [**ATP binding**](http://www.ebi.ac.uk/QuickGO/GTerm?id=GO:0005524)**,** [**motor activity**](http://www.ebi.ac.uk/QuickGO/GTerm?id=GO:0003774) |
| **BAND 12** | | | | | | |
|  | GI:38196217 | transferrin-like, partial (similar to *Anopheles gambiae)* | 7 | 21.4 | 4.67 | ferric iron binding |
| **BAND 19** | | | | | | |
|  | GI:170046511 | Phosphoribosylformylglycinamidine cyclo-ligase (similar to *Culex quinquefasciatus)* | 3 | 99.4 | 7.12 | [ATP binding](http://www.ebi.ac.uk/QuickGO/GTerm?id=GO:0005524), [ligase activity](http://www.ebi.ac.uk/QuickGO/GTerm?id=GO:0004637) |
| **BAND 20** | | | | | | |
|  | GI:167880215 | alpha-glucosidase (similar to *Culex quinquefasciatus)* | 3 | 66.6 | 5.26 | Cation binding |
| **BAND 21** | | | | | | |
|  | **GI:158289831** | **AGAP010479-PA (similar to *Anopheles gambiae*)** | **5** | **45.0** | **6.80** | **Unknown** |
|  | GI:167869463 | conserved hypothetical protein (similar to *Culex quinquefasciatus*) | 45 | 6.5 | 9.17 | Unknown |
| **BAND 24** | | | | | | |
|  | GI:38196187 | SRPN10, partial (similar to *Anopheles gambiae)* | 6 | 21.7 | 8.92 | Serine protease inhibitors |
| **BAND 26** | | | | | | |
|  | GI:1644281 | serine protease (similar to *Anopheles gambiae*) | 4 | 29.2 | 4.87 | Endopeptidase activity |
| **BAND 27** | | | | | | |
|  | GI:108881785 | AAEL002759-PC (similar to *Aedes aegypti*) | 9 | 32.4 | 4.89 | Motor activity |

*Proteins identified common to both in solution and in gel are shown in bold
