# Supplementary material for: Proteome-wide analysis of Anopheles culicifacies mosquito midgut: new insights into the mechanism of refractoriness
Source: BMC Genomics. 2018 May 8;19:337. doi: 10.1186/s12864-018-4729-3 (PMC5941458; doi:10.1186/s12864-018-4729-3)
Supplement: Supplementary file 4 — Table S4. A catalogue of midgut proteins identified using in-gel digestion strategy coupled with LC/MS/MS in An. culicifacies species B. (DOC 97 kb) [file 12864_2018_4729_MOESM4_ESM.doc]

Table S4 A catalogue of midgut proteins identified using in-gel digestion strategy coupled with LC/MS/MS in *An. culicifacies* species B

| **S.no** | **Accession number** | **Protein** | **Seq. Cov** | **M.wt (kDa)** | **C. pI** | **Function** |
| --- | --- | --- | --- | --- | --- | --- |
| **BAND 1** | | | | | | |
| 1. **1** | GI:167881467 | citron ser/thr kinase (similar to *Culex quinquefasciatus*) | 1 | 186.5 | 6.40 | protein kinase activity /ATP binding |
| **BAND 4** | | | | | | |
| 1. **2** | GI:167877984 | glutaminyl-tRNA synthetase (similar to *Culex quinquefasciatus*) | 1.3 | 87.0 | 6.55 | ATP binding |
| **BAND 5** | | | | | | |
|  | GI:167864745 | Guanine nucleotide binding protein, partial (similar to *Culex quinquefasciatus*) | 11 | 36.9 | 9.60 | [metal ion binding](http://www.ebi.ac.uk/QuickGO/GTerm?id=GO:0046872) |
|  | GI:94468818 | heat shock cognate 70 (similar to *Aedes aegypti*) | 2 | 72.2 | 5.20 | Chaperons/ATP binding |
| **BAND 8** | | | | | | |
|  | GI:668461709 | myosin heavy chain, isoform N (similar to *Anopheles sinensis*) | 7 | 215.2 | 6.30 | ATPase activity |
|  | GI:108881785 | AAEL002759-PC (similar to *Aedes aegypti*) | 11 | 32.4 | 4.89 | Motor activity |
|  | GI:118786501 | AGAP005459-PA (similar to *Anopheles gambiae* str. PEST) | 15 | 14.6 | 4.56 | Chitin binding |
| **8** | **GI:118792103** | **AGAP012401-PA (similar to *Anopheles gambiae* str. PEST)** | **7** | **57.2** | **5.74** | **Catalytic activity** |
| **BAND 10** | | | | | | |
| 9 | GI:2654602 | chitinase (similar to *Anopheles gambiae*) | 2 | 57.2 | 5.88 | Chitinase activity |
| **BAND 11** | | | | | | |
| 10 | GI:158297660 | AGAP011453-PA (similar to *Anopheles gambiae*) | 4 | 85.8 | 5.14 | Ferric ion binding |
| **BAND 13** | | | | | | |
| **11** | **GI:158296454** | **AGAP000881-PA (similar to *Anopheles gambiae*)** | **5** | **58.3** | **7.36** | **oxidoreductase activity** |
| **BAND 14** | | | | | | |
| 12 | GI:158300147 | AGAP012407-PA, partial (similar to *Anopheles gambiae*) | 5 | 53.1 | 5.10 | Redox activity |
| **13** | **GI:347970794** | **AGAP003869-PA (similar to *Anopheles gambiae*)** | **2** | **54.0** | **6.73** | **Aminopeptidase activity** |
| **14** | **GI:18389889** | **calreticulin (similar to *Anopheles gambiae*)** | **5** | **46.3** | **4.56** | **Calcium ion binding** |
| **BAND 15** | | | | | | |
| 15 | GI:355398665 | enolase, partial (*similar to* *Aedes albopictus*) | 6 | 20.5 | 5.12 | Not known |
| **BAND 17** | | | | | | |
| 16 | GI:167880215 | alpha-glucosidase (similar to *Culex quinquefasciatus*) | 3 | 66.6 | 5.26 | Cation binding |
| **BAND 18** | | | | | | |
| 17 | GI:301641424 | actin, partial (similar to *Aedes aegypti)* | 34 | 24.0 | 5.07 | Motor activity |
|  | **GI:240270402** | **serpin 10 plasmodium-related inhibitory serine protease inhibitor, partial (similar to *Anopheles gambiae*)** | **10** | **24.5** | **5.31** | **Immune related** |
| **BAND 22** | | | | | | |
|  | **GI:118784691** | **AGAP004571-PA (similar to *Anopheles gambiae*)** | **5** | **35.9** | **5.77** | **serine-type endopeptidase activity** |
| **BAND 25** | | | | | | |
|  | **GI:158299190** | **AGAP010147-PA (similar to *Anopheles gambiae*)** | **7** | **224.2** | **5.76** | **ATPase activity/ATP binding** |
|  | **GI:347964032** | **AGAP000550-PA (similar to *Anopheles gambiae*)** | **3** | **164.1** | **5.52** | **Cell matrix adhesion** |
|  | GI:167862764 | sodium/potassium-transporting ATPase alpha chain (similar to *Culex quinquefasciatus*) | 4 | 80.3 | 5.67 | ATPase activity/ATP binding |
|  | GI:158294268 | AGAP005504-PD (similar to *Anopheles gambiae*) | 4 | 36.4 | 5.15 | Defense against pathogens. |
|  | GI:668459783 | AGAP011476-PA-like protein (similar to *Anopheles sinensis*) | 2 | 99.1 | 5.77 | Unknown |
|  | GI:281186393 | AGAP006342-PA (similar to *Anopheles gambiae*) | 10 | 20.0 | 9.31 | zinc ion binding/amidase acitivity |
|  | GI:119855481 | cytochrome b5 (similar to *Anopheles funestus*) | 12 | 14.7 | 5.17 | Heme binding domains |
|  | GI:118786445 | AGAP005423-PA (similar to *Anopheles gambiae*) | 5 | 27.9 | 8.12 | endopeptidase activity |
|  | **GI:119112622** | **AGAP007790-PA (similar to *Anopheles gambiae*)** | **4** | **35.5** | **8.06** | **ATP ase activity** |
|  | GI:668453876 | glycerol-3-phosphate dehydrogenase (similar to *Anopheles sinensis*) | 4 | 38.2 | 6.92 | oxidoreductase activity |
|  | **GI:668445830** | **AGAP007120-PA-like protein (similar to *Anopheles sinensis*)** | **20** | **17.0** | **7.34** | **kinase activity/ signal transduction** |
|  | GI:158300600 | AGAP012048-PA (similar to *Anopheles gambiae*) | 2 | 51.5 | 8.76 | transferase activity, |
|  | GI:668443881 | AGAP010347-PA-like protein (similar to *Anopheles sinensis*) | 13 | 15.7 | 5.88 | metal ion binding |
|  | **GI:118789564** | **AGAP007963-PA (similar to *Anopheles gambiae*)** | **8** | **22.1** | **4.83** | **calcium ion binding** |
|  | GI:108878479 | AAEL005766-PA (similar to *Aedes aegypti)* | 4 | 39.1 | 7.87 | fructose-bisphosphate aldolase activity |
|  | **GI:58391886** | **AGAP009833-PA (similar to *Anopheles gambiae*)** | **13** | **30.7** | **8.56** | **Transport** |
|  | GI:37703126 | beta-tubulin, partial (similar to *Anopheles gambiae*) | 5 | 20.9 | 5.31 | Polymer forming protein |
|  | GI:167870785 | Histone H4 (similar to *Culex quinquefasciatus*) | 13 | 10.2 | 11.55 | DNA binding |
|  | GI:158299558 | AGAP008909-PA (similar to *Anopheles gambiae*) | 10 | 22.6 | 6.93 | Phosphatidyl Ethanolamine-Binding Protein |

* Proteins identified common to both in solution and in gel are shown in bold
