# Supplementary material for: Proteome-wide analysis of Anopheles culicifacies mosquito midgut: new insights into the mechanism of refractoriness
Source: BMC Genomics. 2018 May 8;19:337. doi: 10.1186/s12864-018-4729-3 (PMC5941458; doi:10.1186/s12864-018-4729-3)
Supplement: Supplementary file 5 — Table S5. A catalogue of identified putative proteins found in both species A and species B of An. culicifacies using iTRAQ labeling method. (DOCX 31 kb) [file 12864_2018_4729_MOESM5_ESM.docx]

Table S5 A catalogue of identified putative proteins found in both Species A and species B of *An. culicifacies* using iTRAQ labeling method

| **S.no** | **Uniprot no.** | **Protein** | **∑ coverage** | **Peptides** | **M.wt (kDa)** | **Function** | **Ratio** |
| --- | --- | --- | --- | --- | --- | --- | --- |
|  | Q8MUR9 | Glutathione S-transferase (similar to *Anopheles gambiae)* | 18 | 4 | 22.3 | Protein binding | 0.782 |
|  | Q7PQK3 | AGAP004212-PA (similar to *Anopheles gambiae* ) | 18 | 11 | 46.3 | [calcium ion binding](http://www.ebi.ac.uk/QuickGO/GTerm?id=GO:0005509) | 0.870 |
|  | T1E826 | Putative adp (similar to *Anopheles aquasalis*) | 16 | 6 | 32.9 | [transporter activity](http://www.ebi.ac.uk/QuickGO/GTerm?id=GO:0005215) | 0.950 |
|  | T1E870 | Putative pdsw (similar to *Anopheles aquasalis*) | 15 | 3 | 19.6 | Not known | 0.755 |
|  | T1DPW3 | Putative actin (Fragment) similar to *Anopheles aquasalis*) | 13 | 6 | 39.7 | [ATP binding](http://www.ebi.ac.uk/QuickGO/GTerm?id=GO:0005524) | 0.701 |
|  | P35035 | Trypsin-1 (similar to *Anopheles gambiae*) | 13 | 3 | 29.0 | [Endopeptidase activity](http://www.ebi.ac.uk/QuickGO/GTerm?id=GO:0004252) | 0.977 |
|  | Q7QE55 | Integrin beta (similar to *Anopheles gambiae*) | 10.99 | 9 | 92.8 | [receptor activity](http://www.ebi.ac.uk/QuickGO/GTerm?id=GO:0004872) | 0.932 |
|  | A7URV6 | AGAP006936-PB (similar to *Anopheles gambiae*) | 10.77 | 3 | 32.7 | electron carrier activity | 0.778 |
|  | P91895 | Triosephosphateisomerase (Fragment) (similar to Anopheles merus) | 10.68 | 2 | 21.9 | [triose-phosphate isomerase activity](http://www.ebi.ac.uk/QuickGO/GTerm?id=GO:0004807) | 0.845 |
|  | O02350 | Zinc carboxypeptidase (similar to *Anopheles gambiae*) | 10.16 | 3 | 49.1 | [metallocarboxy peptidase activity](http://www.ebi.ac.uk/QuickGO/GTerm?id=GO:0004181) | 1.309 |
|  | T1EAU6 | Putative alkaline phosphatase (Fragment) (similar to *Anopheles aquasalis*) | 10.12 | 4 | 36.4 | [phosphatase activity](http://www.ebi.ac.uk/QuickGO/GTerm?id=GO:0016791) |  |
|  | Q7PZ92 | Acyl-coenzyme A oxidase (Fragment) (similar to *Anopheles gambiae*) | 9.48 | 8 | 75.6 | [oxidase activity](http://www.ebi.ac.uk/QuickGO/GTerm?id=GO:0003997) | 0.924 |
|  | Q7QC60 | AGAP002465-PA (similar to *Anopheles gambiae*) | 8.80 | 3 | 24.6 | [ferric iron binding](http://www.ebi.ac.uk/QuickGO/GTerm?id=GO:0008199) | 1.093 |
|  | Q7PXF5 | AGAP001381-PA (similar to *Anopheles gambiae*) | 8.61 | 16 | 212.6 | Laminin N-terminal domain  Interaction | 1.001 |
|  | Q5TWW9 | AGAP007393-PA (similar to *Anopheles gambiae*) | 8.20 | 5 | 54.3 | [oxidoreductase activity](http://www.ebi.ac.uk/QuickGO/GTerm?id=GO:0015035) | 0.719 |
|  | T1E7A2 | Putative mitochondrial phosphate carrier protein (similar to *Anopheles aquasalis*) | 7.02 | 3 | 38.9 | Energy transfer | 1.247 |
|  | Q16N74 | AAEL012062-PA (similar *to Aedes aegypti*) | 6.89 | 8 | 110.9 | [transmembrane transporter activity](http://www.ebi.ac.uk/QuickGO/GTerm?id=GO:0015077) | 0.683 |
|  | T1EA14 | Putative hexokinase (Fragment) (similar to *Anopheles aquasalis*) | 6.74 | 3 | 49.4 | [ATP binding](http://www.ebi.ac.uk/QuickGO/GTerm?id=GO:0005524) | 0.942 |
|  | T1DG61 | Putative superfamily ii dna and rna helicase dna replication (similar to *Anopheles aquasalis*)] | 6.42 | 3 | 45.6 | nucleic acid binding | 1.154 |
|  | B0WIR8 | L(2)37Cc (similar to *Culex quinquefasciatus*) | 6.25 | 2 | 29.8 | Regulation | 1.453 |
|  | Q7Q3J8 | AGAP007918-PA (Fragment) (similar to *Anopheles gambiae*) | 5.79 | 9 | 145.9 | electron carrier activity | 1.386 |
|  | B0W5F2 | ATP synthase subunit alpha (similar to *Culex quinquefasciatus*) | 5 | 4 | 59.3 | [ATP binding](http://www.ebi.ac.uk/QuickGO/GTerm?id=GO:0005524) | 0.900 |
|  | T1DN98 | Putative rna-binding translational regulator irpaconitase superfamily (similar to *Anopheles aquasalis*) | 5.35 | 5 | 85.2 | [Aconitate hydratase activity](http://www.ebi.ac.uk/QuickGO/GTerm?id=GO:0003994) | 0.944 |
|  | P52813 | 40S ribosomal protein S3a (similar to *Anopheles gambiae*) | 5.20 | 2 | 29.8 | [structural constituent of ribosome](http://www.ebi.ac.uk/QuickGO/GTerm?id=GO:0003735) | 1.181 |
|  | A0NBZ0 | AGAP003493-PA (similar to *Anopheles gambiae*) | 4.98 | 3 | 52.2 | [transmembrane transporter activity](http://www.ebi.ac.uk/QuickGO/GTerm?id=GO:0022891) | 0.816 |
|  | Q7QAQ6 | AGAP003581-PA (similar to *Anopheles gambiae*) | 4.96 | 2 | 39.3 | [oxidoreductase activity](http://www.ebi.ac.uk/QuickGO/GTerm?id=GO:0016491) | 1.023 |
|  | T1EB34 | Putative multidrug/pheromone exporter abc superfamily (Fragment) (similar to *Anopheles aquasalis*) | 4.75 | 4 | 81.4 | [ATP binding](http://www.ebi.ac.uk/QuickGO/GTerm?id=GO:0005524) | 0.918 |
|  | Q7Q0Z2 | Beta-hexosaminidase(similar to *Anopheles gambiae*) | 4.15 | 3 | 63.3 | hydrolase activity | 0.938 |
|  | T1DJW4 | Putative udp-glucuronosyltransferase (Fragment) (similar to *Anopheles aquasalis*) | 3.93 | 3 | 57.1 | [transferase activity](http://www.ebi.ac.uk/QuickGO/GTerm?id=GO:0016758) | 0.967 |
|  | Q5TNW4 | AGAP010130-PA (similar to *Anopheles gambiae*) | 3.18 | 1 | 31.6 | catalytic activity | 0.910 |
|  | Q7Q440 | AGAP008193-PA (similar to *Anopheles gambiae*) | 3.13 | 5 | 148 | [calcium ion binding](http://www.ebi.ac.uk/QuickGO/GTerm?id=GO:0005509) | Equal |
|  | B0WTU5 | Coiled-coil domain-containing protein 22 homolog (similar to *Culex quinquefasciatus*) | 2 | 2 | 64.2 | unknown function | 0.797 |
|  | Q5TS29 | AGAP008752-PA (Fragment) (similar to *Anopheles gambiae*) | 2 | 3 | 120.0 | unknown function | 1.354 |
|  | Q16JU2 | AAEL013215-PA (Fragment) (similar to *Aedes aegypti*) | 2 | 6 | 229.6 | [ATP binding](http://www.ebi.ac.uk/QuickGO/GTerm?id=GO:0005524) | 0.667 |
|  | [F5HKJ1](http://mascot/mascot/cgi/protein_view.pl?file=..%2Fdata%2F20150513%2FF001316.dat&hit=F5HKJ1_ANOGA&db_idx=1&px=1&ave_thresh=26&_ignoreionsscorebelow=0&report=0&_sigthreshold=0.05&_msresflags=1097&_msresflags2=2&percolate=0&percolate_rt=0&_minpeplen=7&sessionID=all_secdisabledsession) | AGAP002858-PC (similar to *Anopheles gambiae*) | 4 | 5 | 134.9 | Unknown | 0.74 |
|  | [F1CKA1](http://mascot/mascot/cgi/protein_view.pl?file=..%2Fdata%2F20150513%2FF001316.dat&hit=F1CKA1_ANOAB&db_idx=1&px=1&ave_thresh=26&_ignoreionsscorebelow=0&report=0&_sigthreshold=0.05&_msresflags=1097&_msresflags2=2&percolate=0&percolate_rt=0&_minpeplen=7&sessionID=all_secdisabledsession) | Cytochrome c oxidase subunit 2 (similar to *Anopheles albitarsis)* | 6 | 2 | 27.871 | [cytochrome-c oxidase activity](http://www.ebi.ac.uk/QuickGO/GTerm?id=GO:0004129) | 1.12 |
|  | A0A023EDL7 | Putative ubiquitin/60s ribosomal protein l40 fusion (Fragment) (similar to *Aedes albopictus*) | 26 | 3 | 20.1 | [structural constituent of ribosome](http://www.ebi.ac.uk/QuickGO/GTerm?id=GO:0003735) | 0.73 |
|  | [A0A084VH8](http://mascot/mascot/cgi/protein_view.pl?file=..%2Fdata%2F20150513%2FF001316.dat&hit=A0A084VH89_9DIPT&db_idx=1&px=1&ave_thresh=26&_ignoreionsscorebelow=0&report=0&_sigthreshold=0.05&_msresflags=1097&_msresflags2=2&percolate=0&percolate_rt=0&_minpeplen=7&sessionID=all_secdisabledsession) | AGAP003581-PA-like protein (similar to *Anopheles sinensis*) | 4 | 2 | 46.5 | [oxidoreductase activity](http://www.ebi.ac.uk/QuickGO/GTerm?id=GO:0016491) | 0.9 |
|  | [Q5TWW9](http://mascot/mascot/cgi/protein_view.pl?file=..%2Fdata%2F20150513%2FF001316.dat&hit=Q5TWW9_ANOGA&db_idx=1&px=1&ave_thresh=26&_ignoreionsscorebelow=0&report=0&_sigthreshold=0.05&_msresflags=1097&_msresflags2=2&percolate=0&percolate_rt=0&_minpeplen=7&sessionID=all_secdisabledsession) | Protein disulfide-isomerase (similar to *Anopheles gambiae*) | 5 | 3 | 68.9 | [protein disulfide isomerase activity](http://www.ebi.ac.uk/QuickGO/GTerm?id=GO:0003756) | 0.74 |
|  | [A0A023ETR3](http://mascot/mascot/cgi/protein_view.pl?file=..%2Fdata%2F20150513%2FF001316.dat&hit=A0A023ETR3_AEDAL&db_idx=1&px=1&ave_thresh=26&_ignoreionsscorebelow=0&report=0&_sigthreshold=0.05&_msresflags=1097&_msresflags2=2&percolate=0&percolate_rt=0&_minpeplen=7&sessionID=all_secdisabledsession) | Elongation factor 1-alpha (similar to *Aedes albopictus*) | 3 | 2 | 65.8 | [GTPase activity](http://www.ebi.ac.uk/QuickGO/GTerm?id=GO:0003924) | 1.44 |
|  | [T1DPE1](http://mascot/mascot/cgi/protein_view.pl?file=..%2Fdata%2F20150513%2FF001316.dat&hit=T1DPE1_ANOAQ&db_idx=1&px=1&ave_thresh=26&_ignoreionsscorebelow=0&report=0&_sigthreshold=0.05&_msresflags=1097&_msresflags2=2&percolate=0&percolate_rt=0&_minpeplen=7&sessionID=all_secdisabledsession) | Fructose-bisphosphatealdolase (similar to *Anopheles aquasalis*) | 4 | 2 | 47.4 | [fructose-bisphosphate aldolase activity](http://www.ebi.ac.uk/QuickGO/GTerm?id=GO:0004332) | 0.75 |
|  | [B0WCI6](http://mascot/mascot/cgi/protein_view.pl?file=..%2Fdata%2F20150514%2FF001327.dat&hit=B0WCI6_CULQU&db_idx=1&px=1&ave_thresh=25&_ignoreionsscorebelow=0&report=0&_sigthreshold=0.05&_msresflags=1097&_msresflags2=2&percolate=0&percolate_rt=0&_minpeplen=7&sessionID=all_secdisabledsession) | NADH dehydrogenase (similar to *Culex quinquefasciatus*) | 23 | 4 | 23 | oxidoreductase | 0.73 |
|  | [B0WGV0](http://mascot/mascot/cgi/protein_view.pl?file=..%2Fdata%2F20150514%2FF001327.dat&hit=B0WGV0_CULQU&db_idx=1&px=1&ave_thresh=25&_ignoreionsscorebelow=0&report=0&_sigthreshold=0.05&_msresflags=1097&_msresflags2=2&percolate=0&percolate_rt=0&_minpeplen=7&sessionID=all_secdisabledsession) | Trehalase (similar to *Culex quinquefasciatus*) | 6 | 3 | 81.3 | [alpha,alpha-trehalase activity](http://www.ebi.ac.uk/QuickGO/GTerm?id=GO:0004555) | 0.77 |
|  | [A023EV56](http://mascot/mascot/cgi/protein_view.pl?file=..%2Fdata%2F20150514%2FF001327.dat&hit=A0A023EV56_AEDAL&db_idx=1&px=1&ave_thresh=25&_ignoreionsscorebelow=0&report=0&_sigthreshold=0.05&_msresflags=1097&_msresflags2=2&percolate=0&percolate_rt=0&_minpeplen=7&sessionID=all_secdisabledsession) | Putative transport protein sec61 alpha subunit (similar to Aedes albopictus) | 12 | 6 | 58.2 | [protein transport](http://www.ebi.ac.uk/QuickGO/GTerm?id=GO:0015031) | 0.95 |
|  | [A084W3J5](http://mascot/mascot/cgi/protein_view.pl?file=..%2Fdata%2F20150514%2FF001327.dat&hit=A0A084W3J5_9DIPT&db_idx=1&px=1&ave_thresh=25&_ignoreionsscorebelow=0&report=0&_sigthreshold=0.05&_msresflags=1097&_msresflags2=2&percolate=0&percolate_rt=0&_minpeplen=7&sessionID=all_secdisabledsession) | Hexokinase (similar *to Anopheles sinensis*) | 4 | 2 | 61.6 | [ATP binding](http://www.ebi.ac.uk/QuickGO/GTerm?id=GO:0005524) | 0.95 |
|  | [A084VH94](http://mascot/mascot/cgi/protein_view.pl?file=..%2Fdata%2F20150514%2FF001327.dat&hit=A0A084VH94_9DIPT&db_idx=1&px=1&ave_thresh=25&_ignoreionsscorebelow=0&report=0&_sigthreshold=0.05&_msresflags=1097&_msresflags2=2&percolate=0&percolate_rt=0&_minpeplen=7&sessionID=all_secdisabledsession) | AGAP003586-PA-like protein (similar to *Aedes albopictus*) | 15 | 5 | 46.4 | Unknown | 1 |
|  | [B0XGX7](http://mascot/mascot/cgi/protein_view.pl?file=..%2Fdata%2F20150514%2FF001327.dat&hit=B0XGX7_CULQU&db_idx=1&px=1&ave_thresh=25&_ignoreionsscorebelow=0&report=0&_sigthreshold=0.05&_msresflags=1097&_msresflags2=2&percolate=0&percolate_rt=0&_minpeplen=7&sessionID=all_secdisabledsession) | Annexin (similar to *Culex quinquefasciatus*) | 14 | 7 | 42.8 | [calcium ion binding](http://www.ebi.ac.uk/QuickGO/GTerm?id=GO:0005509) | 0.9 |
|  | Q1HQX4 | Transmembrane trafficking protein (similar to *Aedes aegypti*) | 22.6 | 4 | 23.8 | Protein transport | 0.88 |
|  | Q179E8 | AAEL005656-PA (similar to *Aedes aegypti*) | 22 | 53 | 221.3 |  | 0.82 |
|  | Q9BMG6 | Putative transport protein Sec61 alpha subunit (similar to *Aedes aegypti*) | 12.6 | 7 | 52.1 | Protein transport | 0.853 |
|  | Q7Q7M8 | AGAP004593-PA (similar to *Anopheles gambiae*) | 12.5 | 7 | 61.4 | Calcium ion binding | 0.66 |
|  | Q16M31 | AAEL012439-PA (similar to *Aedes aegypti*) | 12.3 | 3 | 35.1 | Catalytic activity | 0.739 |
|  | Q7QJW0 | AGAP007684-PA (similar to *Anopheles gambiae*) | 9.7 | 4 | 52.7 | Cysteine type Peptidase activity | 1.123 |
|  | A7UVP0  (SP 1-21) | AGAP001346-PA (similar to *Anopheles gambiae*) | 8.53 | 2 | 22.7 | unknown | 0.739 |
|  | Q7Q0S1 | AGAP010191-PA (Fragment) (similar to *Anopheles gambiae*) | 8.3 | 3 | 39.0 | Calcium ion binding | 0.742 |
|  | Q5TW92 | AGAP001037-PA (similar to *Anopheles gambiae*) | 7.75 | 4 | 68.0 | Metal ion binding/metalloaminopeptidase activity | 0.951 |
|  | Q1HQU9  (1-22) | AAEL010065-PA (similar to *Aedes aegypti*) | 6.86 | 4 | 47.6 | Isomerase activity/Thioredoxin domain | 1.380 |
|  | Q7Q4I1 | AGAP008408-PA (similar to *Anopheles gambiae*) | 6.67 | 4 | 66.1 | Protein binding | 0.775 |
|  | Q175A3 | AAEL006721-PA (similar to *Aedes aegypti*) | 6.43 | 7 | 118.5 | Oxoglutarate dehydrogenase activity | 0.935 |
|  | T1DNF9 | Putative metalloexopeptidase (similar to *Anopheles* aquasalis) | 5.80 | 4 | 53.8 | metallopeptidase activity | 0.737 |
|  | Q7PWT2 | AGAP008861-PA (similar to *Anopheles gambiae*) | 5.79 | 2 | 27.5 | Serine type endopeptidase activity | 0.794 |
|  | Q7QDZ4 | AGAP010733-PA (Fragment) (similar to *Anopheles gambiae*) | 5.68 | 4 | 56.2 | Hydrolase activity | 0.930 |
|  | Q7QFD4 | AGAP000416-PA (similar to *Anopheles gambiae*) | 5.66 | 2 | 29.4 | hydroxyacylglutathione hydrolase activity | 0.758 |
|  | T1DJC8 | Putative vacuolar h+-atpase v0 sector subunit d (Fragment) (similar to *Anopheles* aquasalis) | 5.65 | 2 | 38.3 | hydrogen ion transmembrane transporter activity | 0.902 |
|  | Q7PRI8 | AGAP010383-PA (Fragment) (similar to *Anopheles gambiae*) | 5.48 | 5 | 78.7 | transporter activity | 0.787 |
|  | Q17BB4 | AAEL005037-PA (similar to *Aedes aegypti*) | 5.40 | 3 | 55.9 | nucleotide binding/ aminoacyl-tRNA ligase activity | 1.489 |
|  | B0WQT6 | Serrate protein (similar to *Culex quinquefasciatus*) | 5.36 | 20 | 409.6 | calcium ion binding / protein binding | 1.422 |
|  | B0X0A3 | Coatomer subunit gamma (similar to *Culex quinquefasciatus*) | 5.35 | 6 | 93.6 | structural molecule activity | 1.060 |
|  | M4K018 | LANB2 (similar to *Anopheles gambiae*) | 4.99 | 9 | 179.5 | protein binding | 0.923 |
|  | Q7Q6C1 | AGAP005929-PA (similar to *Anopheles gambiae*) | 4.85 | 2 | 34.1 | pyridoxal kinase activity | 0.889 |
|  | B1Q287 | AGAP002919-PA (similar to *Anopheles gambiae*) | 4.83 | 2 | 48.0 | structural constituent of ribosome | 1.351 |
|  | J9HYY6 | AAEL017004-PA (similar to *Aedes aegypti*) | 4.50 | 4 | 75.8 | thiol-dependent ubiquitinyl hydrolase activity/protein binding | 0.779 |
|  | Q7QC14 | AGAP002399-PA (similar to *Anopheles gambiae*) | 4.21 | 3 | 40.3 | Unknown | 0.950 |
|  | B0WJL7 | Cap-G (similar to *Culex quinquefasciatus*) | 4.06 | 9 | 167.8 | binding | 0.727 |
|  | B0WAI2 | Angiotensin-converting enzyme (similar to *Culex quinquefasciatus*) | 3.99 | 6 | 142.8 | metallopeptidase activity | 0.897 |
|  | Q7QJQ1 | AGAP007612-PA (similar to *Anopheles gambiae*) | 3.76 | 4 | 92.1 | Unknown | 0.821 |
|  | A7UU61 | AGAP006224-PA (similar to *Anopheles gambiae*) | 3.39 | 5 | 138.7 | oxidoreductase activity/  catalytic activity | 0.816 |
|  | F5HLF2 | AGAP013393-PA (similar to *Anopheles gambiae*) | 3.28 | 4 | 104.1 | metallopeptidase activity | 0.827 |
|  | Q7PXW9 | AGAP001633-PA (similar to *Anopheles gambiae*) | 2.79 | 26 | 913.6 | protein kinase activity | 0.988 |
|  | Q5TS29 | AGAP008752-PA (Fragment) (similar to *Anopheles gambiae*) | 2.71 | 3 | 120.0 | Unknown | 1.395 |
|  | T1DJH7 | Putative elongation factor 2 (Fragment) (similar to *Anopheles aquasalis*) | 2.49 | 3 | 94.3 | GTPase activity | 0.903 |
|  | Q5TWN6 | AGAP007061-PA (similar to *Anopheles gambiae*) | 2 | 3 | 138.1 | protein binding | 1.123 |
